# Subfossil Leaves Reveal a New Upland Hardwood Component of the Pre-European Piedmont Landscape, Lancaster County, Pennsylvania

**DOI:** 10.1371/journal.pone.0079317

**Published:** 2013-11-13

**Authors:** Sara J. Elliott, Peter Wilf, Robert C. Walter, Dorothy J. Merritts

**Affiliations:** 1 Department of Geosciences, Pennsylvania State University, University Park, Pennsylvania, United States of America; 2 Department of Earth and Environment, Franklin and Marshall College, Lancaster, Pennsylvania, United States of America; The Pennsylvania State University, United States of America

## Abstract

Widespread deforestation, agriculture, and construction of milldams by European settlers greatly influenced valley-bottom stream morphology and riparian vegetation in the northeastern USA. The former broad, tussock-sedge wetlands with small, anastomosing channels were converted into today’s incised, meandering streams with unstable banks that support mostly weedy, invasive vegetation. Vast accumulations of fine-grained “legacy” sediments that blanket the regional valley-bottom Piedmont landscape now are being reworked from stream banks, significantly impairing the ecological health of downstream water bodies, most notably the Chesapeake Bay. However, potential restoration is impaired by lack of direct knowledge of the pre-settlement riparian and upslope floral ecosystems. We studied the subfossil leaf flora of Denlingers Mill, an obsolete (breached) milldam site in southeastern Pennsylvania that exhibits a modern secondary forest growing atop thin soils, above bedrock outcrops immediately adjacent to a modified, incised stream channel. Presumably, an overhanging old-growth forest also existed on this substrate until the early 1700s and was responsible for depositing exceptionally preserved, minimally transported subfossil leaves into hydric soil strata, which immediately underlie post-European settlement legacy sediments. We interpret the eleven identified species of the subfossil assemblage to primarily represent a previously unknown, upland Red Oak-American Beech mixed hardwood forest. Some elements also appear to belong to a valley-margin Red Maple-Black Ash swamp forest, consistent with preliminary data from a nearby site. Thus, our results add significantly to a more complete understanding of the pre-European settlement landscape, especially of the hardwood tree flora. Compared with the modern forest, it is apparent that both lowland and upslope forests in the region have been modified significantly by historical activities. Our study underscores that generally overlooked subfossil leaves can provide important, local, temporally constrained paleoecological data, with much potential value in this case for stream and wetland restoration decisions in the mid-Atlantic region.

## Introduction

Post European-settlement activities, including land clearing, agriculture, and milldam construction, rapidly and significantly modified Piedmont stream hydrology and riparian forest ecology and composition in eastern North America [1]–[4]. The secondary successional forests that dominate the landscape today do not provide the same ecological functionality as the ancient, pre-settlement forests [5]. Moreover, detrimental consequences of these modifications to water quality and aquatic habitats, including eutrophication and loss of biodiversity, have been evident for decades in vital downstream water bodies such as the ecologically, commercially, and recreationally important Chesapeake Bay, the largest estuary in the United States [6]. Plant macrofossils can yield significant insights into local pre-colonial communities, contributing critical knowledge to the overall understanding of regional historical landscape modifications. Additionally, a precise representation of the pre-settlement flora can enhance the success of future stream, wetland, and floodplain rehabilitation because plant assemblages directly affect fluvial and wetland hydrology, geomorphology, and bank stability [7].

Recent studies have used sedimentological and paleontological data to characterize the impacts of European settlement on wetland and riparian vegetation in the northeastern U.S.A. Fossil fruits and seeds recovered from the bases of stream cutbanks in southeastern Pennsylvania and Maryland indicate that prior to widespread post-settlement activities, valley bottoms contained palustrine wetlands with small, shallow channels, interconnected pools, and herbaceous obligate wetland species [4], [8], [9]. These wetland-meadow communities were composed mainly of *Carex* spp. (sedges, including *C. prasina, C. crinita, C. stipata, C. stricta*), as well as *Polygonum* spp. (knotweeds), *Eleocharis* spp. (spikerushes), and *Scirpus* spp. (bulrushes). Among woody plants, only a few shrubs and occasional trees from areas fringing the wetlands were recognized initially, including *Liriodendron tulipifera* (Tulip Tree) and *Juglans cinerea* (Butternut) [8]. Although it appears that valley bottom wetlands were widespread by the mid-Holocene, dating of the recovered fossil seeds indicates that a patchwork of wetland environments may have existed from ca. 11,240 ybp [4] until the arrival of European settlers approximately 300 years ago [10]. A single, unpublished study of leaf macrofossils from southeastern Pennsylvania provided evidence of woody, riparian wetland and non-wetland species within this system [11], [12]. However, leaf preservation was fragmentary, and data from non-wetland trees were especially limited.

Plant macrofossils are ubiquitous within organic-rich deposits from river and floodplain environments [13]–[15]. Fossils discovered within stream cutbanks have been used successfully as valuable sources of Pleistocene and Holocene paleoecological information worldwide [15]–[18]. However, most Holocene analyses have been based on wood, seeds, fruits, and pollen because identifiable leaves are typically less abundant than these other organs at any particular locality [19], and they are also fragile and difficult to process. As has long been known in deep-time paleoecology, leaf-dominated assemblages are produced via fundamentally different taphonomic pathways from fruit and seed assemblages and generally are transported shorter distances, providing much more localized data [19]–[23]. Moreover, leaves are minimally time-averaged because of their general inability to be reworked, which results in much higher temporal resolution than seed, fruit, and pollen deposits, usually on the scale of 10^1^ to 10^3^ years [19], [20], [23]. When used together with other macrofossil and palynological data, fossil leaves can greatly increase the accuracy of paleobotanical reconstructions [19].

### Ecological Effects of Prehistoric and Historic Land Use

Beginning with Native American influences, the Piedmont regions of the United States have a long history of anthropogenic landscape modifications, which greatly increased due to the colonial-era conversions of millions of acres of old-growth forest into agricultural fields during the 17^th^ through 19^th^ centuries [24]. These areas supplied agricultural goods and other commodities to colonial port cities for trade [25]. The mid-Atlantic Piedmont region was the wheat belt for the early colonies. Certain crops, particularly tobacco, were highly detrimental to soil quality, causing settlers to abandon fields and clear additional land every few seasons [1]. It has been recognized since the 1700s that these extensive clearing and tilling practices led to large-scale regional topsoil erosion from ridge tops and other elevated areas down into Piedmont valley bottoms, eventually causing sedimentation onto wetlands, streams, floodplains, and valley margins [24], [26].

Furthermore, expansive milldam construction in valley bottoms amplified the influences of upland deforestation and denudation, causing extreme shifts in the regional hydrologic, geomorphologic, and riparian vegetation regimes [4]. Sedimentological data indicate that the mid-Atlantic Piedmont area was altered especially due to the markedly high density of constructed dams [4], [8]. United States census data show that no fewer than 65,000 water-powered mills were operating in the eastern U.S. by 1840, at least 10,000 of which were in Pennsylvania by the mid 19^th^ century [4]. Of these, an estimated 450+ mills were built in Lancaster County alone by 1850 (Barton Collection, Lancaster County Historical Society), averaging to one dam per every two km of stream length [4].

Milldams stretched across entire valley bottoms and were used primarily to power mills, forges, furnaces, and mining operations. This extensive framework significantly decreased flow velocity, causing suspended sediments to settle out of the water column and to fill the millpond reservoirs located directly upstream of dams. Because dams were spaced so closely together, the lacustrine deposits behind one usually stretched upstream to the next. The intensive dam infrastructure caused a regional elevation of the Piedmont floodplain surface relative to hydrologic base level [4], ultimately burying the pre-existing, anabranching channel valley-bottom floodplain system [27]. The upland-derived, fine-grained, nutrient-rich muds and silts that accumulated behind dams, in stream channels, and on valley-bottom floodplain wetlands due to centuries of intensive land use by Euro-Americans are collectively known as “legacy sediments” [4]. Eventual breaching and/or intentional removal of dams led to new incision of channels into the accumulated reservoir deposits and the exposure, erosion, and redistribution of legacy sediments to downstream waterways. As a result, the regional hydrogeomorphology of first-to-third order streams shifted, from small, vegetated, anastomosing channels that were well-connected with the water table and their floodplains, into the steep-banked, single-channel meandering streams seen in valley bottoms today [4], [27], [28], [29].

Due to the dramatic rise in the regional floodplain surface relative to the groundwater table, it became increasingly difficult for historic vegetation to access sufficient water. Thus, modifications to hydrogeomorphology effectively transformed the riparian buffer into one dominated at present by a weedy assemblage of thistles and grasses capable of surviving on the functionally mesic to xeric legacy sediments, such as *Agropyron repens* (Quackgrass) [4], [8]. Declining water levels after dam breaching and incision also exposed nutrient-rich sediment surfaces that had been previously inundated [4], [30], [31]. Therefore, in addition to the initial stage of replacement by weedy vegetation, disturbance-adapted invasive species such as *Cirsium arvense* (Canada Thistle) and *Dactylis glomerata* (Orchard Grass) began to outcompete native vegetation through rapid growth, high fecundity, and efficient dispersal mechanisms [32]–[34].

The vegetation shifts produced secondary valley margin and upslope forests that differed in structure, composition, and function from their pre-settlement counterparts. For instance, successional forests typically have lower frequencies of snags, fewer layers of foliage and age classes of trees, simpler canopy gap structure [35], and significantly lower accumulations of aboveground living and dead tree biomass [5]. They also fail to produce ecologically important in-stream habitat characteristics such as debris dams and associated pools [5], [36]. Thus, in addition to the decreases in hyporheic exchange and biogeochemical reaction rates caused by modification of stream morphology and floodplain elevation, historical alterations of riparian buffers have negatively impacted forest ecosystem functions [27].

Native American populations also affected Piedmont riparian floodplains, including hydrological and ecological alterations similar to those of colonial Europeans, but they did so on a much smaller scale. Archaeological evidence, charcoal, and isotopic analyses suggest expansive historical forest clearing and use of fire by East Coast Native American tribes [37]–[39], as well as decreases in old-growth forest taxa and increases in crop, disturbance, and early successional species [40], [41]. One study of Delaware River flood deposits indicated that maize agriculture substantially decreased upland forest cover and caused subsequent erosion and sedimentation in valley bottoms as early as 1000 C.E. [42]. However, the magnitude of impact was significantly less than that of the 17^th^–19^th^ century European settlers [42], [43]. Therefore, the attributes of the pre-European landscape determined through sedimentological and paleontological analyses can be used as historical reference baselines, with the caveat that pre-settlement conditions were not pristine sensu stricto, simply much less degraded than now [44], [45].

### Stream Restoration

Renewed efforts in recent years to reconstruct the pre-European fluvial and vegetative landscape [4], [8], [9], [29], [46]–[48] were initiated by observations of anomalously high erosion rates to downstream watersheds of ca. 50–400 times the long-term regional geologic average [49]. A recent effort to quantify the floodplain and bank sediment budget for a 28 km stretch of the Little Conestoga Creek, a high sediment-yielding component of the Chesapeake Bay Watershed, showed a net mean sediment loss, indicating that contemporary bank erosion of historic legacy sediments exceeds modern-day floodplain sedimentation [50]. Many studies have concluded that modern topsoil erosion certainly contributes to the sediment flux [37], [51], but it is increasingly understood that more than half of the suspended sediment load currently carried by Piedmont rivers and streams is derived from the erosion of channel-bank legacy sediments, rather than contemporary soil erosion from upland farms and development sites [46], [52], [53]. Furthermore, it has been shown that instead of quickly abating, reservoir deposits behind milldams can continue to be sources of fine-grained sediment for at least several decades after breaching [53]. Therefore, the construction and demise of milldams have been important influences on fluvial and erosional processes in the region, and, ultimately, these historical activities are probably more responsible than modern agricultural practices for the high suspended sediment loads that negatively affect modern downstream ecosystems such as the Chesapeake Bay [53], [54].

Past stream restoration efforts in the Piedmont have shown limited success when the effects of historical sediment alteration were not considered [34]. These approaches focused on “restoring” stream channels, without consideration of surrounding floodplains and sediment deposits, and ultimately compounded the issues faced by downstream ecosystems without improving ecological services and functions [4], [27]. Recently, it has been demonstrated that a more sustainable and effective restoration method is to remove legacy sediments completely and to restore the naturally occurring riparian vegetation at groundwater level wherever possible [8], [27], [34]. Holocene wetland soils buried by legacy sediments preserve a detailed paleo-seed record of the stable valley-bottom plant communities that existed for thousands of years prior to European settlement [4], [8], [10]. The identification of these buried seeds can be used to construct a “seed library” to determine the most appropriate species to plant following restoration [8], [27], especially in the absence of many studies that document vegetation colonization or succession following dam removals in the region [33]. However, one study researching ecological responses to small dam removals in the Midwestern USA showed that pioneer communities consist of grasses and forbs, and that it can take upwards of three decades for hardwood trees to naturally colonize disturbed riparian zones [55].

Efforts to quantify the number and distribution of river restoration projects in the United States have revealed a distinct lack of written records detailing project outcomes [56]. This lack of post-restoration monitoring has led to the recent notion that the act of restoration itself has outpaced our scientific investigation and understanding [57]. For example, despite extensive restoration efforts, it remains unclear whether the health of Chesapeake Bay has broadly improved with regards to its water quality and biodiversity [57].

Legacy sediments in the study region contain large concentrations of total nitrogen (ca. 1600 mg/kg) and phosphorus (ca. 600 mg/kg), which can contribute to high nutrient loading in downstream ecosystems via erosion of stream banks [46]. This high nutrient content causes enrichment in rivers, lakes, and coastal areas [58], and many negative effects of both pulsed and sustained inputs of sediments to stream biota are documented [59], [60]. From an ecological perspective, for instance, high nutrient content in surface waters leads to eutrophication, i.e. the enhancement of phytoplankton productivity due to nutrient enrichments, which eventually can cause hypoxic conditions. In general, phytoplankton growth is limited by the availability of P in freshwater systems and is N limited in marine systems. The Pennsylvania Chesapeake Bay Tributary Strategy recognizes P as the limiting nutrient in the State’s surface waters and that efforts to reduce P concentrations are tangibly linked to reducing sediment loads [46].

The characteristics of Piedmont streams within the Chesapeake watershed caused them to become the largest sources of fine-grained, suspended sediment into the Chesapeake Bay [47]. As a result, the Bay has a well-documented history of algal blooms, eutrophication, and hypoxic and anoxic conditions [4], [61], [62]. Population declines of many ecologically important organisms have been observed for decades, including sea grasses and water-filtering oysters, all at least partially caused by eutrophication [6], [63] stemming from the influx of nutrient-laden legacy sediment. An accurate representation of both the pre-settlement valley bottom vegetation and the transitional valley margin and upslope hardwood tree flora is beneficial to stream restoration because riparian buffer vegetation is considered one of the most important drivers in restoration project success [64]. This is, in part, due to the numerous positive ecological and economic benefits provided by a functioning riparian buffer, including increased channel stability, decreased sediment and nutrient loading both locally and downstream, increased groundwater recharge and hyporheic exchanges, and increased healthy habitat for important stream and wetland biota [27], [34].

### Importance of the Study Site and Goals of the Research

It is reasonable to expect that not only the valley-bottom wetland and riparian vegetation described in previous preliminary work [8], but also the non-wetland transitional and upslope forest vegetation has been altered dramatically since European settlement. The goals of this study are to provide the first significant insights into the pre-settlement non-wetland to upslope (upland) hardwood tree assemblages that grew adjacent to the stream-filled valley bottoms, and to lend further accuracy to the regional pre-settlement, old-growth forest framework. Possible implications include aiding restoration and conservation efforts as well as historic understanding of colonial-era forests.

The deposition of large, concentrated, and generally unabraded leaves at Denlingers Mill in Lancaster County, Pennsylvania (see Methods) indicates that abundant subfossil leaf material was shed locally, minimally transported, sorted, or reworked hydrodynamically, and deposited shortly after entering the stream-wetland network [14], [65]–[67]. Our preliminary regional observations are that preserved leaf mat layers occur rarely compared to the more ubiquitous and widespread Holocene wetland soils that contain the sedge-dominated paleo-seed record, and that all leaf mat layers occur near valley margins and/or are located directly upstream of milldam sites. The leaf mat exposure used in this study is exceptionally prolific and informative due to elevated local bedrock spurs located immediately adjacent to the channel. The spur on the north side of the stream, ca. 7–8 m high, today supports a forest of tall-canopied trees that overhang and deposit large numbers of leaves into the channel. Because the Mid-Atlantic region is tectonically inactive (except for post-glacial isostatic adjustments) and has low, positive relief, this subdued upland landscape presumably would have supported large overhanging trees on the same bedrock, contributing prodigious quantities of leaves to the pre-settlement wetland channels and floodplain, as today’s forest in the same location does to the present altered stream. The leaf macrofossils from Denlingers Mill, therefore, provide both a novel, non-wetland signal for the pre-settlement valley-margin and upslope forests, and an opportunity to directly compare the old-growth pre-settlement forest composition to the altered secondary forest that grows on the same substrate today.

## Methods

### Ethics Statement

No permits were required for the described study, which complied with all relevant regulations.

### Site Description and Stratigraphy

**Figure 1:**
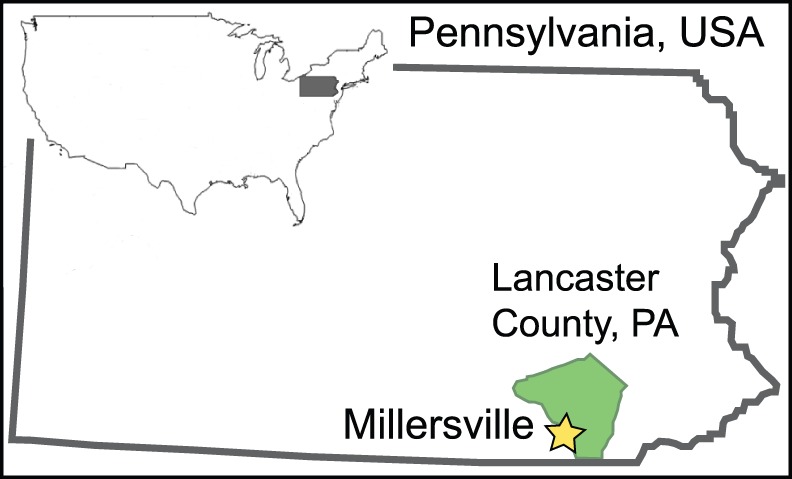
Location of the Denlingers Mill subfossil site. West Branch of the Little Conestoga Creek near Millersville, in Lancaster County, Pennsylvania, USA.

**Figure 2:**
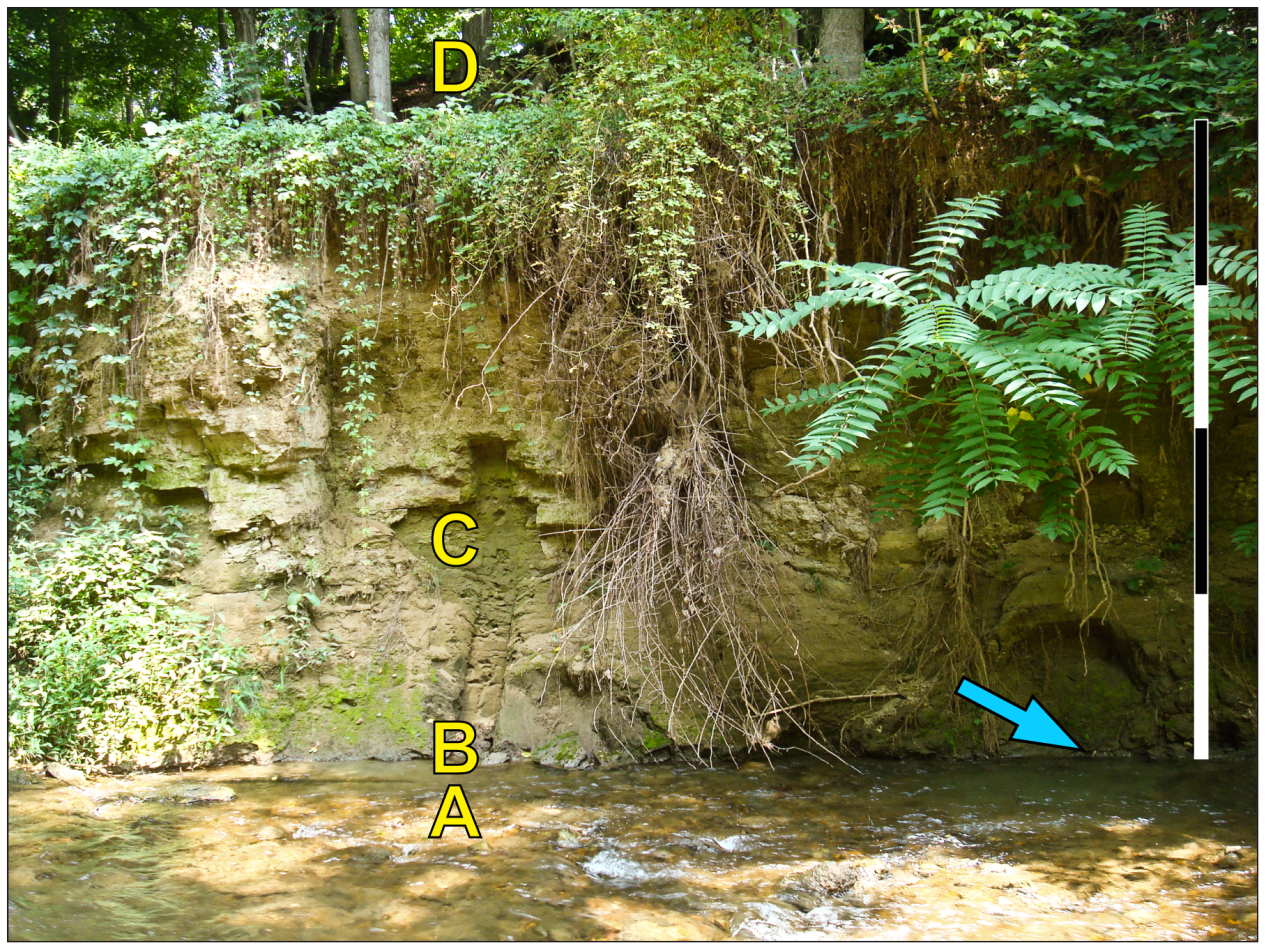
Denlingers Mill leaf mat site (arrow). (A) Limestone and phyllite bedrock and quartz gravel composing channel bed. (B) Darker hydric soil layer containing plant macrofossils. (C) Approximately 4 m of silty legacy sediment. (D) Exposed bedrock supporting a contemporary riparian forest. Arrow points to exposure from which all subfossils for this study were collected. Each scale bar unit = 1 m. See also Figure 3.

Denlingers Mill (DM) is located on the West Branch of Little Conestoga Creek, in Lancaster County, Pennsylvania, 5 km southwest of Millersville (fossil site location 39°58″27.11′ N, 76°22″33.33′ W; Figs. 1, 2). Lancaster County is part of the Piedmont physiographic province within the Appalachian Highlands, characterized by broad rolling hills and valleys with underlying Ordovician Conestoga Limestone in the northern and central parts and the lower Paleozoic Marburg (formerly Wissahickon) Schist in the western and southern parts of the county [52], [68]. Bedrock at DM consists of Conestoga Limestone, and personal observations of outcrops indicate that limestone with phyllitic partings, in particular, directly surrounds the sampled area.

The West Branch of Little Conestoga Creek is a second-order stream within the Conestoga River watershed, which feeds the Susquehanna River and ultimately the Chesapeake Bay. This stream is approximately 10 km long, and historical evidence suggests that a milldam was built every ca. 1 km along its length [52]. According to historical accounts, the dam at DM was built in the early 1700s, was ca. 5 m high, trapped sediment in a pond that extended at least 800 m upstream (terminating at the next milldam), and eventually breached sometime during the early 20^th^ century [46], [48].

**Figure 3:**
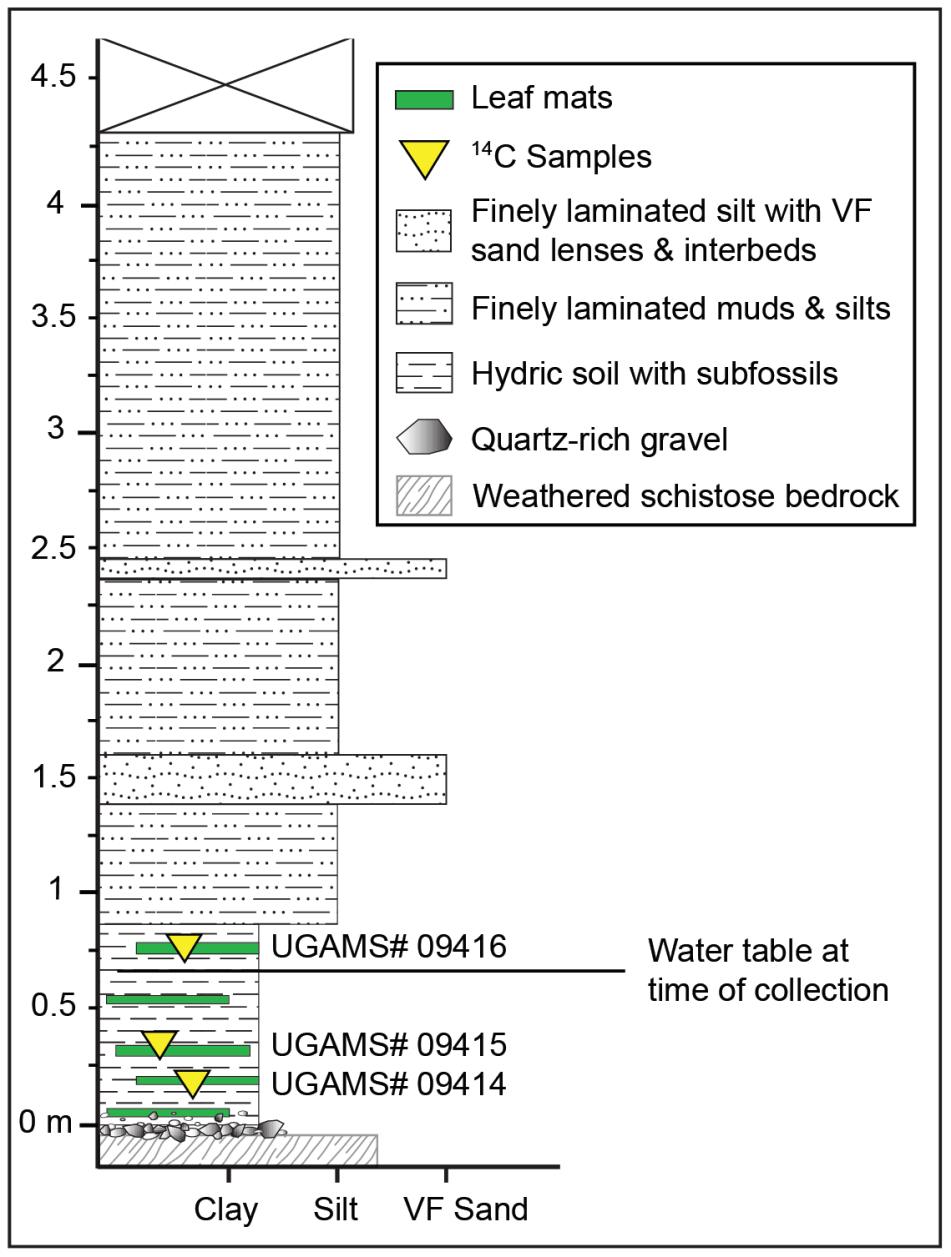
Stratigraphic profile of the Denlingers Mill leaf mat site. Green blocks indicate the presence of dense leaf mat layers within the hydric soil unit. Subfossil leaves in this study were taken from all leaf mat layers. Yellow triangles indicate locations of samples taken for ^14^C dating. The X at the top of the section represents the covered interval. See also Figure 2.

The Denlingers Mill stratigraphic profile was measured and described at the centimeter scale during May, 2011 (Fig. 3). To ensure accuracy of descriptions, most overhanging vegetation on the profile was cleared, and a fresh vertical surface was exposed on a small section of the stream bank adjacent to the subfossil exposure. The entire stratigraphic section up to the base of the exposed upland (upslope) bedrock is approximately 4.2 m thick and closely parallels the regional composite profile of mid-Atlantic streams described by Walter and Merritts [4], as follows.

Beginning at the base of the exposure, limestone bedrock with phyllitic partings is overlain by angular to sub-rounded quartz gravels (Basal Gravels in Fig. 3), above which is a black hydric soil ca. 80 cm thick (Holocene wetland). Regionally, this hydric soil layer contains pedologic features such as root structures and plant macrofossils [8] and has been dated by ^14^C to range from ca. 11,500–300 ybp, terminating at European settlement [4]. The hydric soil exposure at DM is located approximately 20 m upstream from the old milldam wall structure and 110 meters upstream from the confluence with the Little Conestoga Creek. The top of the hydric soil is dense with leaf mats, seeds, twigs, and remnant herbaceous material, and it was the source of all subfossils used in this study. The remaining 3.6 meters above the hydric soil is composed of finely laminated legacy muds and silts with thin, very fine sand interbeds and lenses. Hanging roots and weedy vegetation obscured the uppermost portion of the profile.

Three leaf fragments were sent to the Center for Applied Isotope Studies at the University of Georgia for accelerator mass spectrometry (AMS) radiocarbon dating (Table 1). The “basal” and “middle” samples were taken from the (typically) submerged leaf mat layers, and the “top” sample was taken from the upper portion of the hydric soil unit (Fig. 3). The returned ages in radiocarbon years BP were then calibrated using the CalPal online radiocarbon calibration package [69].

**Table 1:** Radiocarbon ages of leaf macrofossils from Denlingers Mill.

Sample Number	Years BP	Cal AD
UGAMS 09414 (DM#1– Basal)	110±20	1806 CE ±94
UGAMS 09415 (DM#3– Middle)	100±20	1805 CE ±94
UGAMS 09416 (DM#5– Top)	150±20	1810 CE ±113

*Footnotes*: In uncalibrated years BP & calibrated calendar years AD; analytical uncertainties of calibrated ages ±2 sigma. Calibrated ages obtained using CalPal 2007 online radiocarbon calibration package [69] available: http://www.calpal-online.de/. Samples were analyzed for accelerator mass spectrometry (AMS) radiocarbon dating at the Center for Applied Isotope Studies, University of Georgia.

**Figure 4:**
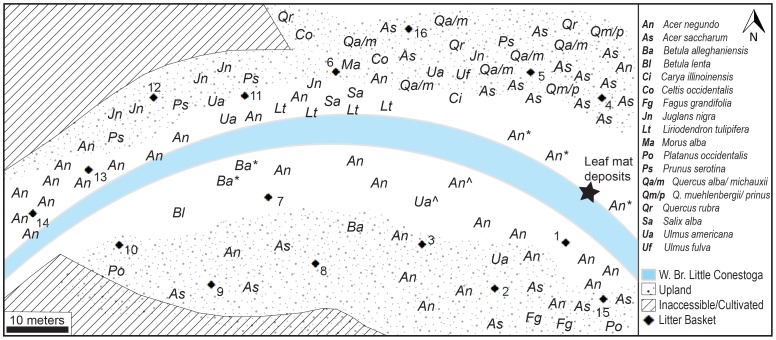
Schematic diagram of 50× 100 m half-hectare plot in the contemporary riparian and upslope forest. Diamonds denote the 16 leaf litter collection baskets. The relative locations of all identified trees within the plot are coded by the first letters of the genus and species. There were a total of 92 trees >10 cm dbh. Codes followed by an asterisk (*) were not counted in the stand summary because their stems were <10 cm dbh. Codes followed by a caret (ˆ) denote trees that fell due to flooding from Hurricane Irene in the fall of 2011, and were also not counted. All species are native to North America except *Morus alba*, which is native to China.

Denlingers Mill is unusual compared to other legacy sediment sites studied so far because it has upland bedrock spurs that allow a secondary riparian forest corridor to grow adjacent to the stream. This strip of contemporary forest, dominated at present by *Acer negundo* (Box Elder) and *Acer saccharum* (Sugar Maple), is only ca. 100 m wide because of contiguous cultivated and developed land. *Acer negundo* individuals grow adjacent to the measured area, and contemporary leaf litter from a variety of hardwood species is abundant in modern point bar deposits and within the channel (Fig. 4; Tables 2, 3). The limestone bedrock spur has phyllitic partings, is weathered (saprolitized), and tapers in the northeasterly direction above the subfossil exposure.

**Table 2:** Denlingers Mill half-hectare stand. Summary of the 18 identified species, listed in abundance order.

Species	#Stems	R S #	T B A (m^2^)	R B A
Box Elder (*Acer negundo*)	30	32.61	4.95	35.68
Sugar Maple (*Acer saccharum*)	20	21.74	2.33	16.81
White Oaks (*Q. alba/Q. michauxii*)	6	6.52	0.30	2.00
Walnut (*Juglans nigra*)	6	6.52	0.35	2.53
Black Cherry (*Prunus serotina)*	4	4.35	0.33	2.41
Tulip (*Liriodendron tulipifera*)	4	4.35	0.60	4.31
American Elm (*Ulmus americana*)	4	4.35	0.88	6.34
Red Oak (*Quercus rubra*)	3	3.26	0.45	3.25
Chinkapin Oak (*Q. muehlenbergii)*	2	2.17	0.40	2.92
American Beech (*Fagus grandifolia*)	2	2.17	0.05	0.33
Sycamore (*Platanus occidentalis*)	2	2.17	1.50	10.83
Hackberry (*Celtis* sp.)	2	2.17	0.42	3.06
White Willow (*Salix alba*)	2	2.17	0.75	5.43
Yellow Birch (*Betula alleghaniensis*)	1	1.09	0.15	1.08
Sweet (Black) Birch (*Betula lenta*)	1	1.09	0.10	0.72
Pecan (*Carya illinoinensis*)	1	1.09	0.17	1.20
Slippery Elm (*Ulmus fulva*)	1	1.09	0.09	0.62
White Mulberry (*Morus alba*)	1	1.09	0.07	0.48
**Totals**	**92**	**100**	**13.89**	**100**

*Footnotes*: Data from 92 stems >10 cm in diameter were counted. R S # = Relative Stem Number. T B A = Total Basal Area in meters squared. R B A = Relative Basal Area.

**Table 3:** Summary of the 23 identified modern leaf litter species at Denlingers Mill.

Species	Frequency	M L #	% L #	M L W	% L W
*Acer negundo*	93.30	23.73	43.89	3.00	29.20
*Acer saccharum*	80.00	10.53	19.44	1.91	18.60
*Q. alba/Q. michauxii*	20.00	0.27	0.49	0.06	0.60
*Juglans nigra*	6.70	0.33	0.62	0.05	0.47
*Prunus serotina*	40.00	1.33	2.47	0.32	3.06
*Liriodendron tulipifera*	46.70	2.93	5.43	1.62	15.69
*Ulmus americana*	40.00	2.47	4.57	0.78	7.57
*Quercus rubra*	53.30	2.33	4.31	1.00	9.74
*Q. muehlenbergii/Q. prinus*	20.00	0.60	1.11	0.19	1.88
*Fagus grandifolia*	13.30	0.20	0.37	0.03	0.27
*Platanus occidentalis*	20.00	0.67	1.24	0.71	6.86
*Celtis occidentalis*	0.00	0.00	0.00	0.00	0.00
*Salix alba*	13.30	1.20	2.22	0.03	0.31
*Betula alleghaniensis*	33.30	0.87	1.61	0.09	0.90
*Betula lenta*	20.00	0.53	0.99	0.06	0.58
*Carya illinoinensis*	20.00	2.33	4.32	0.11	1.11
*Ulmus fulva*	6.70	0.07	0.12	0.04	0.36
*Morus alba*	0.00	0.00	0.00	0.00	0.00
*Carya glabra*	26.70	3.00	5.56	0.23	2.23
Unidentified *Carya* sp.	13.30	0.20	0.37	0.02	0.19
*Alnus serrulata*	6.70	0.07	0.07	0.01	0.08
*Nyssa Sylvatica*	6.70	0.27	0.49	0.01	0.14
Unidentified *Prunus* sp.	6.70	0.07	0.12	0.09	0.18
**Totals**		**54.0**	**100.00**	**10.36**	**100.00**

*Footnotes:* ML# = Mean Leaf Number. % L # = Percent Leaf Number. M L W = Mean Leaf Weight. % L W = Percent Leaf Weight.

The wedge of alluvial sediment buildup in a dam reservoir is typically thickest near the dam structure itself, where water is deepest and velocity lowest [48]. The DM exposure is located at the downstream end of the former slackwater pond, producing the lowest potential for erosion and thus the greatest potential for preserving underlying leaf deposits. Because DM contains an exceptionally thick package of legacy sediment upstream of the milldam remains (Figs. 2, 3), but not downstream, it was among the first derelict dam sites recognized and studied sedimentologically in the region [52]. Previously recognized *in situ* tree stumps and carbonized organic debris layers below the legacy sediment deposits along the West Branch of the Little Conestoga Creek upstream of DM were originally interpreted as remnants of the forest floor and riverine wetlands that predated colonial settlement [52]; however, the current interpretation is that the valley bottom was a mosaic of ecosystems dominated by a tussock-sedge wet meadow [4], [8], [10]. These organic-rich layers [52] also contain the abundant leaf macrofossils studied here.

### Sample Collection and Processing

Samples were collected from the DM leaf-mat site five times between December, 2010 and October, 2011. Numerous wet blocks of the hydric soil layer (approximately 0.08 m^3^ total; see Stratigraphy) were carefully removed from the exposure using a trowel. Portions of each hydric soil block that were evidently rich in leaves and leaf fragments were extracted, stored, and transported in stream water, within either five-gallon buckets or airtight Ziploc® containers.

Methods for cleaning and mounting were modified from Miller [12]. On arrival in the Penn State Paleobotany and Sedimentology laboratories, all samples were placed in a 50–50 solution of ethanol and high-purity deionized water. Individual leaves, leaf fragments, and occasional fruits were removed from the leaf mat layers by hand, using metal spatulas, soft-bristled paintbrushes, and occasionally a weak solution of hydrogen peroxide, although this tended to damage leaf surfaces. Samples were placed in a 40% HCl bath (approximate pH of 1) for three to five days to remove adhered clay and colloidal material. Organic debris was then removed by soaking leaves in 5% KOH for 5–15 minutes; additional time tended to destroy leaf tissue. After rinsing with high-purity deionized water, samples underwent a final brushing until the leaf surfaces were between 90–100 percent clean of debris. Generally, this procedure did not need to be repeated.

An alternative method using hydrofluoric acid was attempted. Although HF significantly reduced the time and effort needed for cleaning specimens, managing the samples during the HF bath proved unfeasible because the fragile leaves tended to become damaged or disjointed within the small HF-rated containers, providing no benefit to compensate for the increased safety risk.

Immediately after cleaning, samples were dehydrated over the course of one week in a graded series of ethanol baths (50%, 75%, and 100%), then placed in a xylene bath for 5–10 minutes. Following dehydration, leaves were mounted using Cytoseal 280 High Viscosity Mounting Medium (Richard Allen Scientific Inc., Kalamazoo, Michigan, USA) on glass microscope slides of varying sizes, up to 4”×6” for the largest leaves (Ted Pella Inc., Redding, California, USA), and secured with cover glasses. Before receiving specimens, slides were warmed for 2–3 minutes on a hot plate to minimize the quantity of air bubbles trapped in the viscous mounting medium, although it proved impossible to eliminate all air bubbles due to the irregular leaf thicknesses. Leaf specimens were positioned with abaxial sides facing up, to highlight venation and to increase the likelihood of observing stomata and trichomes. These methods preferentially recovered leaves of woody plants, the focus of this study, and a few relatively large fruits. The sediment also contains fragmentary leaves and stems of herbaceous, probably monocot, wetland plants; in all likelihood, it also preserves smaller plant material such as fruits, seeds, palynomorphs, etc. as seen at a nearby site [9]. A total of 108 specimens were identified at least to genus in this study; 104 are mounted leaf specimens, and the remaining four are macrofossil fruits and seeds. All specimens are deposited at the Earth and Mineral Sciences Museum, Pennsylvania State University (EMS; Table S1).

Due to the extensive amount of time required for each sample preparation, limiting the number of slides that could be mounted, statistical quantification of the subfossil assemblage was not feasible. Instead, presence/absence and qualitative relative abundance data were collected. Actualistic studies such as those of Burnham [14] and Burnham et al. [67] show that in temperate deciduous forests containing approximately twenty arborescent species per hectare, 12–15 leaf litter collections spaced at canopy height or less will capture at least 70% of species, usually only excluding rare species. It also has been documented that fossil leaf assemblages are likely to occur in approximately correct rank abundances [23]. Therefore, it is reasonable to infer that the sampled portion of the fossil deposit at DM captured the dominant pre-settlement floodplain and upland tree species, potentially in accurate rank abundances.

### Imaging and Identification

A Nikon D90 camera and Hakuba KLV-700 Lightviewer 7000 Pro were used for macrophotography of all mounted samples and reference samples. Mounted samples were further examined using transmitted and epifluorescent light microscopy, on a Nikon SMZ-1500 stereomicroscope and a Nikon LV100 compound microscope, sharing an EXFO X-Cite 120 epifluorescence illumination unit with an Endow GFP Longpass Emission green filter (Chroma Technology, Number 41018, exciter HQ470/40x, dichroic Q49LP BS, emitter HQ500LP). A Nikon DSRi1 CCD microscope camera and Nikon NIS Elements v.3 Basic Research Software were used for microphotography. Composite photographs generated from images taken at multiple focal planes were assembled using the Align and Blend tools in Adobe Photoshop CS5 (Adobe Systems Incorporated, San Jose, California, USA). Standard, reversible, whole-image adjustments to exposure, contrast, and white balance were performed using Adobe Camera Raw CS5. All images were also checked for artifacts from blends and adjustments, and none were apparent.

Methods outlined in *The Manual of Leaf Architecture* [70] were used to describe the characteristic leaf traits of individual specimens, including venation patterns, margin type, and tooth shape. These descriptions were eventually pooled for recognized species (see Systematics). Because leaves were generally incomplete, not all characters could be determined. Preliminary identifications were based on reference images and general morphological features described from many sources, including PW’s York County and Allegheny National Forest, Pennsylvania leaf collections [71] and cleared leaf images from the Jack A. Wolfe USGS Cleared Leaf Collection, housed at the Smithsonian Institution National Museum of Natural History, Division of Paleobotany.

Identifications based on morphology and leaf architecture using the sources listed above were followed by more rigorous comparisons, taking into account epidermal features such as foliar trichomes and stomatal configurations. Trichome types were characterized using the nomenclature from the *Atlas of foliar surface features in woody plants I*, *VIII*, and *IX* [72]–[74]. Especially within family Betulaceae and the genus *Quercus*, certain trichome types (such as multiradiate and stellate) seemed to be preferentially lost before or during deposition, although all trichomes seemed to be lost from *Quercus* samples. Therefore, when using foliar trichomes to identify species, the absence of specific trichome types was noted but not relied on. When possible, cuticle characters were also compared using images from the Cuticle Database Project [75].

Given the low likelihood that the pre-settlement assemblage contained non-native or invasive species, only those species historically native to the northeastern U.S.A. were considered here. Because the leaf-mat layers were probably deposited during the Little Ice Age (1550–1850 CE; see Results), during which time the average global climate cooled by 0.6°C [76], past species ranges may have differed from modern bounds.

### Modern Forest Study and Leaf Litter Collection

In order to compare the modern forest to the subfossil assemblage, a contemporary stand and leaf litter study was conducted near the end of maximum leaf fall, during late October, 2011. Using methods modified from Burnham et al. [67], a half-hectare was measured and mapped around the Denlingers Mill fossil site, extending 50 m across the stream and 100 m upstream (Fig. 4). Due to cultivated and agricultural land abutting the riparian zone on both sides of the stream, this was the largest possible mappable area at the site. Within the plot, all stems >10 cm dbh (1.4 m) were measured for circumference and identified to species. Relative abundance and total stem basal area data were calculated for all identified hardwood species within the plot (Table 2).

Additionally, a leaf litter summary was conducted for comparison to, and quantification of, the contemporary stand assemblage. As per Burnham et al. [67], 16 wooden bushel baskets were placed evenly around the site at a distance less than or equal to the height of the canopy, i.e., ca. 15–25 m apart during October, 2011 (Fig. 4). The baskets collected leaf litter fall for one week; attempts to collect litter in the stream over the course of the same week were unsuccessful. The data from one basket (9) were unusable because the basket overturned during the collection period. Litter was collected, sorted, counted, and weighed by species (Table 3). Some leaf litter, particularly that of white oaks, was not clearly identifiable to species due to the degree of degradation; therefore, representative specimens of white oaks were lumped into two groups. Based on the general morphology of the decomposing leaves, and the standing species observed in the plot, “White Oak group 1” is most likely *Quercus alba* and/or *Q. michauxii*, whereas “White Oak group 2” is most likely composed of *Q. prinus* and/or *Q. muehlenbergii* (Fig. 4).

**Figure 5:**
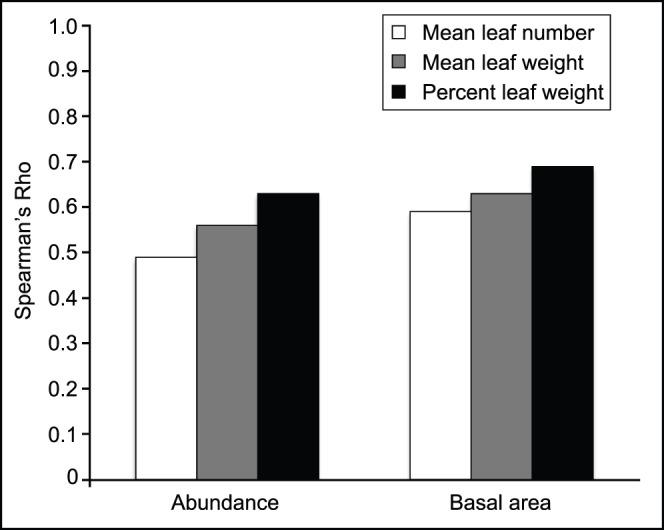
Histogram showing positively correlated rho values for all contemporary forest data. For both abundance and basal area (m), rho is highest for percent leaf weight (i.e., leaf biomass).

Spearman’s rank-order correlations were calculated using the R statistical package [77] in order to compare the stand and leaf litter data (Table 4, Fig. 5), which were arcsine-transformed for percentage data and log_10_-transformed for all other data. Spearman’s rho values were tested for statistical significance using standard critical value tables and graphs. With df = 21, *P*-values were calculated using the AS89 algorithm, specifically designed for Spearman’s rank-order correlation data within the R statistical package. All methods indicated at least 99% confidence for all rho values.

**Table 4:** Spearman’s rank order correlations between modern stand and leaf litter data.

Transformed Data	Rho	Significance(99% confidence)
Abundance vs. mean leaf #	0.49	*P* = 0.0089
Abundance vs. mean leaf weight	0.56	*P* = 0.0031
Abundance vs. percent leaf weight	0.63	*P* = 0.0007
Basal area vs. mean leaf number	0.59	*P* = 0.0014
Basal area vs. mean leaf weight	0.63	*P* = 0.0009
Basal area vs. percent leaf weight	0.69	*P* = 0.0002

## Results

### Radiocarbon Dating

Based on the three analyzed radiocarbon dated leaf fragments, the leaf mat at DM accumulated in the hydric soil between ca. 1697–1899 C.E., including two sigma uncertainties for calibrated ages (Table 1). The samples have the same calibrated age and showed no associated floral differences, and thus all floral samples were pooled and analyzed as a single unit. These radiocarbon ages are consistent with historical data that indicate that construction of the dam, whose trapped sediments postdated and entombed the fossil deposit, occurred sometime during the early 18^th^ century [46], [48]. Thus, the fossils are in all likelihood from the older part of their radiocarbon age range (i.e., early 1700s). Similar hydric soil layers in the region have been ^14^C dated as far back as ca. 11,500 ybp [4], but given the comparatively recent ^14^C age range of these leaves, from the top of the buried hydric soil, we consider the subfossil species assemblage identified from DM to be representative of the last local old-growth forests.

### Contemporary Riparian Forest Composition

A total of eighteen standing species were identified in the half-hectare sample (Tables 2, 4). Of these, two species of maple, *Acer negundo* and *A. saccharum*, clearly dominated the assemblage in terms of abundance and basal area. Interestingly, the leaf litter collection captured 88% of species identified in the stand data, as well as five additional hardwood species not seen in the stand. These included two species of *Carya* (hickories), *Alnus serrulata* (Hazel Alder), *Nyssa sylvatica* (Black Tupelo), and *Prunus* sp. (cherry; Table 3). Spearman’s Rho was highest between percent leaf weight (leaf biomass), and both stem abundance and stem basal area, similar to earlier studies [67]. The strong correlations between the stand and leaf litter data indicate that both provide an accurate portrayal of modern day species richness and abundance at the site (Table 4).

### Denlingers Mill Fossil Flora

Eleven species or morphotypes of hardwood trees were identified from preserved leaves and occasional fruits. All species in the subfossil assemblage are currently native to Pennsylvania and classified as one of the following: facultative upland, meaning they occur in non-wetland settings 67–99% of the time; facultative, meaning they are equally likely to occur in wetlands or non-wetlands; facultative wetland, meaning they inhabit wetlands 67–99% of the time; or upland, indicating no significant occurrence in wetland settings [78].

ORDER Fagales.

FAMILY Betulaceae.

GENUS *Betula* L.

SPECIES *Betula lenta* L.

### Referred Material

**Figure 6:**
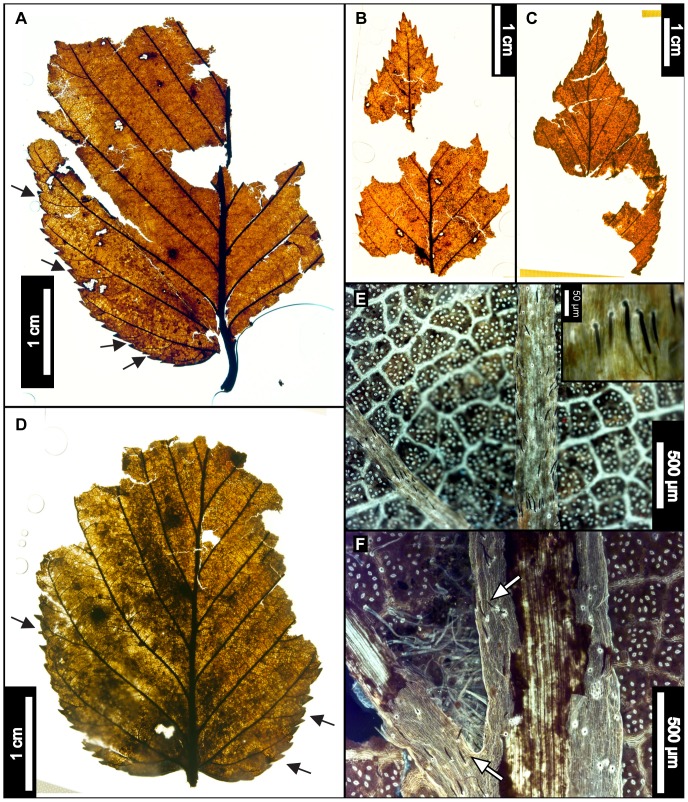
*Betula lenta* (Sweet Birch). (A) Whole subfossil EMS 419583, showing doubly serrated margin. Arrows point to compound agrophic veins. (B) EMS 419582, showing acute apex and doubly serrated margin. Also note the insect damage on (A) and (B). (C) EMS 419581, exhibiting a doubly serrated margin and an acuminate apex. (D) Whole subfossil EMS 419583, showing cordate base, doubly serrated margin, and compound agrophic veins (arrows). (E) EMS 419581, cuticular and stomatal configuration. Epifluorescence image. Inset image is a closeup of subulate trichomes from EMS 419578. (F) Axillary trichomes from EMS 419583. Subulate trichomes also evident on primary and secondary veins (arrows). Epifluorescence image.

EMS 419578– EMS 419592 (Fig. 6).

#### Relevant distinguishing features

*Betula lenta* (Sweet Birch) leaves are ovate or elliptic, with a finely, sharply, singly or doubly toothed margin, a round or cordate base, and an acute to acuminate apex [79], [80]. Sweet Birch has 9–12 pairs of secondary veins and compound agrophic veins that branch near the leaf margin [81]; it tends to be pubescent on the petiole and on veins and vein junctions of the abaxial surface [74], [79], [82]. This species commonly exhibits short, stiff, tapered subulate trichomes (30–100 µm), characteristic of family Betulaceae, along the midvein and secondary veins [74].

#### Description

Laminar sizes of the five scored subfossils range from nanophyll to mesophyll, after extrapolating for the missing leaf areas. The laminae are elliptic in shape and show medial and basal symmetry with a marginal petiole attachment. Apices are either straight and acute, or acuminate. All preserved bases are cordate, with an obtuse basal angle. Naked basal secondary veins are sometimes present. Laminar surfaces are pubescent and sometimes have scattered surficial glands. Margins are sharply serrate, with angular sinuses, and either one order of regularly spaced teeth, or two orders of irregularly spaced teeth that can be crowded and overlapping. Depending on the laminar size, there are 4–7 teeth/cm. Common tooth shapes (apical/basal) are flexuous/flexuous, flexuous/convex, straight/convex, straight/flexuous, convex/convex, convex/straight, concave/convex, concave/flexuous, and, rarely, flexuous/retroflexed. Principal veins terminate at tooth apices, accompanied by straight or concave accessory veins. Primary venation is pinnate, with three basal veins. Compound agrophic veins are present. Major secondaries are craspedodromous to semicraspedodromous, with regular and uniform spacing. Secondary veins are mainly excurrent on the midvein, but they can become decurrent towards the leaf base. Costal secondary veins exhibit a smoothly increasing proximal vein angle. Minor secondary veins are usually craspedodromous but can be semicraspedodromous. Intercostal tertiary veins are sinuous to convex opposite percurrent, or mixed percurrent. They are obtuse to the midvein and have a proximally increasing vein angle. Intercostal tertiaries can decrease in vein angle exmedially. Epimedial tertiaries are opposite, alternate, or mixed percurrent. Proximal vein course is usually perpendicular to the midvein, but it can also be slightly obtuse or parallel to the intercostal tertiaries. The distal course of the epimedial tertiaries is parallel to the intercostal tertiaries. Most exterior tertiary veins terminate at the margin, although some terminate at the sinus. Quaternary and quinternary vein fabrics are both irregular reticulate. Sixth order veins are random reticulate. Freely ending veinlets are absent, and areolation exhibits good development. Subulate trichomes are evident on almost all primary and secondary veins of all specimens. Solitary and acicular trichomes are evident fringing the midveins, as well as in primary and secondary vein axils. Rarely, single trichomes can be seen scattered on the laminar surfaces.

#### Remarks

Within the living regional flora, *Betula lenta* leaves most closely resemble those of *Betula alleghaniensis* (Yellow Birch). Typically, these species are most easily differentiated by bark characteristics [80], [83], [84], and thus identification based on subfossil leaves alone was difficult. However, the fossils exhibit features not seen in leaves of *B. alleghaniensis,* including compound agrophic veins (Fig. 6A, D), a puberulent cuticle surface (Fig. 6E), subulate trichomes on costal veins (Fig. 6E, F), and dense patches of trichomes only in vein junctions (Fig. 6F) [74], [80], [83]. The fossils were therefore classified as *Betula lenta*.

The Sweet Birch is native to the northeastern United States, occurring from southern Maine, west to Ohio and Kentucky, and south to Alabama. Isolated populations also occur in southern Quebec and southeastern Ontario [80]. Sweet Birch is an aromatic tree between 15 and 24 m tall, with a rounded crown of spreading branches. This species prefers moist upland forests, usually along streams and on rocky slopes, and commonly forms associations with other hardwoods and with conifers [80], [83]. *Betula lenta* is classified as a facultative upland species [78].

GENUS *Ostrya* Scop.

SPECIES *Ostrya virginiana* (Mill.) K. Koch.

### Referred Material

**Figure 7:**
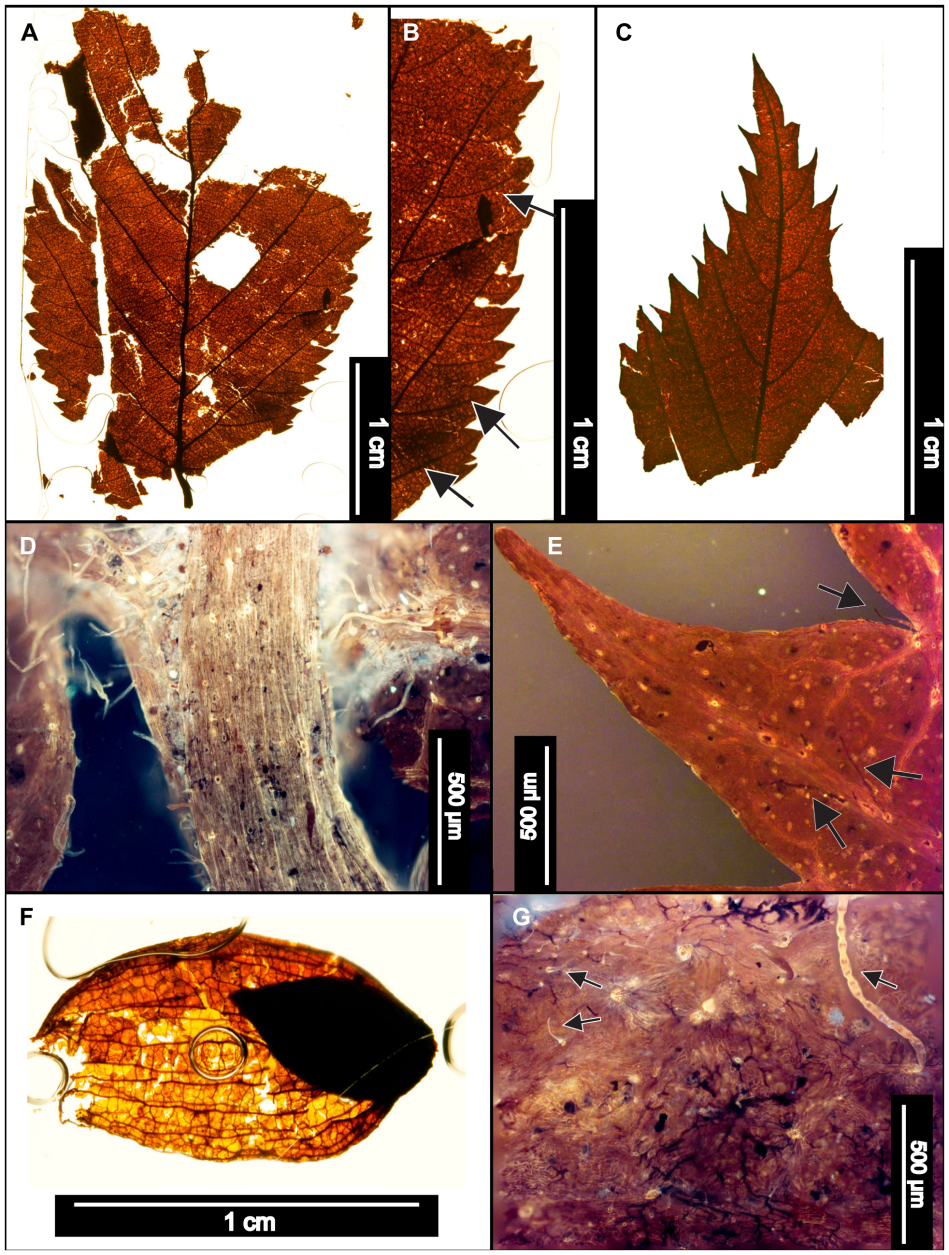
*Ostrya virginiana* (Eastern Hophornbeam). (A) Whole subfossil EMS 419594, exhibiting straight parallel secondary veins. (B) Zoom of EMS 419594, doubly serrated margin and simple agrophic veins (arrows). (C) Whole subfossil EMS 419593, showing acuminate apex with doubly serrated margin. (D) Pubescent petiole and marginal trichomes of EMS 419594. Epifluorescence image. (E) Arrows point to acicular trichomes on vein and subulate trichome in marginal sinus on EMS 419593. Epifluorescence image. (F) EMS 419595, fruit with enclosed seed. (G) Arrows point to various trichome types on fruit (EMS 419595). Epifluorescence image.

EMS 419593– EMS 419595 (Fig. 7).

#### Relevant distinguishing features

*Ostrya virginiana* (Eastern Hophornbeam) leaves are distinguished by a sharply, unevenly, doubly serrated margin, an abruptly acuminate apex, and many nearly straight, parallel secondary veins [80]. The petioles of *O. virginiana* are short and pubescent, and abaxial cuticle surfaces exhibit subulate and acicular trichomes along veins and leaf margins [74]. Primary surface relief is formed by distinctly convex or dome-shaped epidermal cells [74].

*Ostrya virginiana* fruits form involucres up to 5 cm long [80]. Individual seeds are acute, cuspidate, 0.6 cm long nutlets within inflated, papery, pubescent bracts [79], [80], [83]. The bracts have finely reticulated higher-order veins between primary parallel veins and are also villous, exhibiting bristly trichomes around the base and on primary veins [79].

#### Description

Two leaf subfossils were recovered: one apex and one microphyll-sized lamina that is elliptic in shape with medial and basal symmetry and has a pubescent petiole with a marginal blade attachment. The base angle is obtuse, and the shape is potentially convex or cordate but is not fully preserved. The apex is acuminate. Laminar surfaces are pubescent, with dense, scattered surficial glands. The margin is irregularly doubly serrate with angular sinuses and 7–10 teeth/cm. Principal tooth shapes include flexuous/retroflexed, flexuous/flexuous, straight/flexuous, and concave/flexuous. The principal veins terminate at tooth apices, and accessory veins are straight or concave. The primary venation is pinnate, with three basal veins. Simple agrophic veins are present. Major secondary veins are craspedodromous and have uniform vein angles. Secondary spacing is regular but can slightly decrease proximally. Intercostal tertiary vein fabric is mixed percurrent, with veins ranging from straight, convex, and sinuous, to alternate with regular offsets. Tertiaries are obtuse to the midvein and can either maintain a consistent vein angle or slightly increase proximally. Epimedial tertiaries are opposite percurrent to mixed percurrent. The proximal course is either slightly obtuse or perpendicular to the midvein, whereas the distal course becomes parallel to the intercostal tertiaries. Exterior tertiary veins terminate at the margin. Quaternary and quinternary vein fabrics are irregular reticulate. Freely ending veinlets are absent, and areolation exhibits moderate to good development. Small acicular trichomes can be seen on margins of both subfossils. Longer solitary trichomes are evident on costal veins on the abaxial surface.

The preserved seed (nutlet) is 0.6 cm long, within a 1.4 cm involucre. The bracts exhibit many long parallel veins connected by a smaller-gauge, reticulated vein network. Trichomes are also evident on the veins of the bracts (Fig. 7G).

#### Remarks

Within the regional flora, *Ostrya virginiana* (Eastern Hophornbeam) is most similar to *Carpinus caroliniana* (Eastern Hornbeam), from which it is usually distinguished by its shaggy bark and distinctive fruit involucres [79], [84]. However, even though the leaves of these two species are especially similar, the abaxial surfaces of *Carpinus caroliniana* laminae are considerably less pubescent than those of *O. virginiana* [79], [81]. The fossils exhibit straight parallel secondary veins and simple agrophic veins (Fig.7A, B), a doubly serrate margin (Fig. 7A–C), a pubescent petiole (Fig. 7D), and marginal trichomes, as well as long solitary trichomes on veins (Fig. 7D, E). The leaf fossils were classified as *Ostrya virginiana* due to the combination of these features, especially the pubescent petiole and high density of trichomes on veins and margins.

The isolated fruit (EMS 419595) reinforces this conclusion because it is a parallel-veined, thin, papery involucre (Fig. 7F) exhibiting various types of trichomes (Fig. 7G), whereas *C. caroliniana* fruits are ribbed nutlets surrounded by 3-lobed leafy bracts [82] that were not found in our sample.

*Ostrya virginiana* is native to North America, ranging from southeast Manitoba to northern Florida, and west through eastern Texas [80]. The Eastern Hophornbeam usually grows between 6–15 m tall, sometimes reaching heights of 20 m [85], and forms a rounded crown of slender, spreading branches [80]. This species typically inhabits moist soils in the understories of upland hardwood forests [80], but it also frequents dry, wooded slopes, often on calcareous soils [81]. *Ostrya virginiana* is considered a facultative upland species [78].

FAMILY *Fagaceae.*

GENUS *Castanea* Mill.

SPECIES *Castanea dentata* (Marsh.) Borkh.

### Referred Material

**Figure 8:**
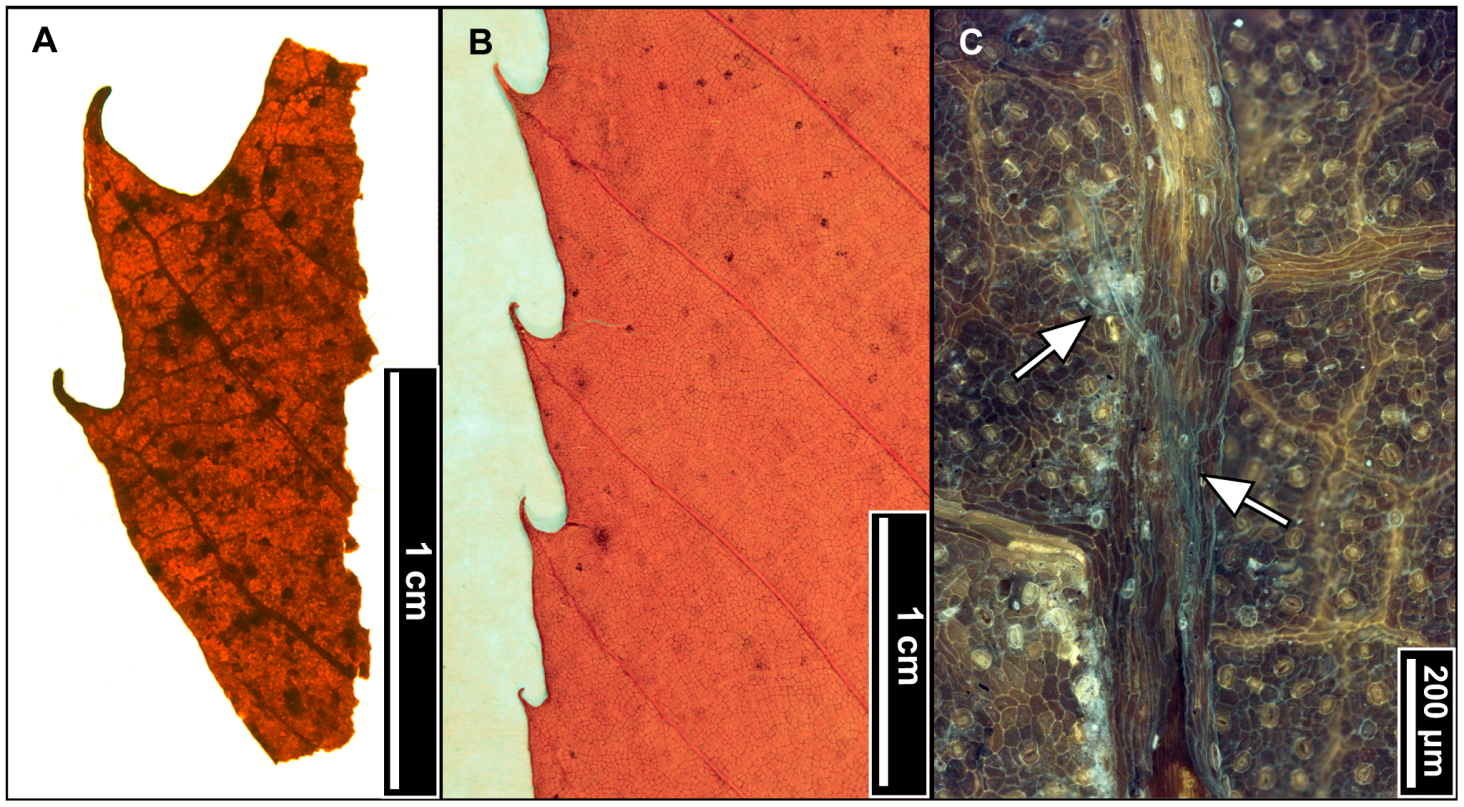
*Castanea dentata* (American Chestnut). (A) Whole subfossil EMS 419555, showing margin with hooked teeth (B) *Castanea dentata* reference image from sample Y1–4, York County, Pennsylvania [72]. (C) EMS 419555, epifluorescence image showing protruding epidermal cells. Arrows point to solitary trichomes on veins.

EMS 419555 (Fig. 8).

#### Relevant distinguishing features

*Castanea dentata* (American Chestnut) leaves are typically oblong and have distinctly curved or hooked, awned teeth with deeply rounded sinuses [80], [86]. The teeth are regularly spaced, each occurring at the end of a straight, parallel secondary vein [80]. Diagnostic cuticle features include protruding epidermal cell walls and a puberulent abaxial leaf surface exhibiting solitary, fasciculate, bulbous, and other trichome types [73]. Trichomes occur on costal veins and sometimes on margins [73].

#### Description

The single subfossil is a marginal fragment approximately 1.5 cm long, and therefore many characters could not be scored. The margin is serrate, with deep rounded sinuses. Tooth spacing is regular, with 2 teeth/cm. Tooth shape is apically concave and basally retroflexed, creating a characteristically hooked appearance. Principal veins terminate in tooth apices. The laminar surface texture is fairly smooth, with only a few trichomes on or near the veins. Primary venation is not visible. Major secondaries are craspedodromous. Intercostal tertiary veins are straight or sinuous opposite percurrent; there are some alternate percurrent veins exmedially. Exterior tertiary veins terminate at the margin. Quaternary and quinternary vein fabrics are both regular reticulate. Freely ending veinlets are absent, and areolation exhibits good development.

#### Remarks

The American Chestnut closely resembles other members of Fagaceae, including *Quercus muehlenbergii* (Chinkapin Oak) and *Castanea pumila* (Allegheny Chinkapin), but it is distinguishable based on leaf morphology and tooth characteristics. Compared to the American Chestnut, leaves of the Chinkapin Oak are typically obovate, with wavy edges and rounder teeth [80]. Similarly, Allegheny Chinkapin leaves have coarser, more irregularly toothed margins, and are tomentose on the abaxial cuticle surface [73], [82]. The subfossil exhibits both characteristic awned, hooked teeth at the ends of parallel veins (Fig. 8A) and solitary, clearly not tomentose trichomes on veins (Fig. 8C), and it is therefore classified as *Castanea dentata*.

This species is native to the northeastern United States and was historically ubiquitous and dominant, ranging from southern Ontario and Maine, south to Florida, and west to Mississippi and Indiana [78], [80]. The American Chestnut has a massive trunk and naturally grows upwards of 30 m, forming a broad, rounded crown. Due to the effects of the Chestnut Blight fungus introduced to North America at the turn of the 20^th^ century, any wild trees today are usually small, multi-stemmed resprouts, ca. 5–10 m tall, that emerge from the living root systems of dead trunks [80], [86]. The American Chestnut prefers moist upland soils in mixed forests [80], and, because it is an upland species, it is not assigned a wetland indicator status [78].

GENUS *Fagus* L.

SPECIES *Fagus grandifolia* Ehrh.

### Referred Material

**Figure 9:**
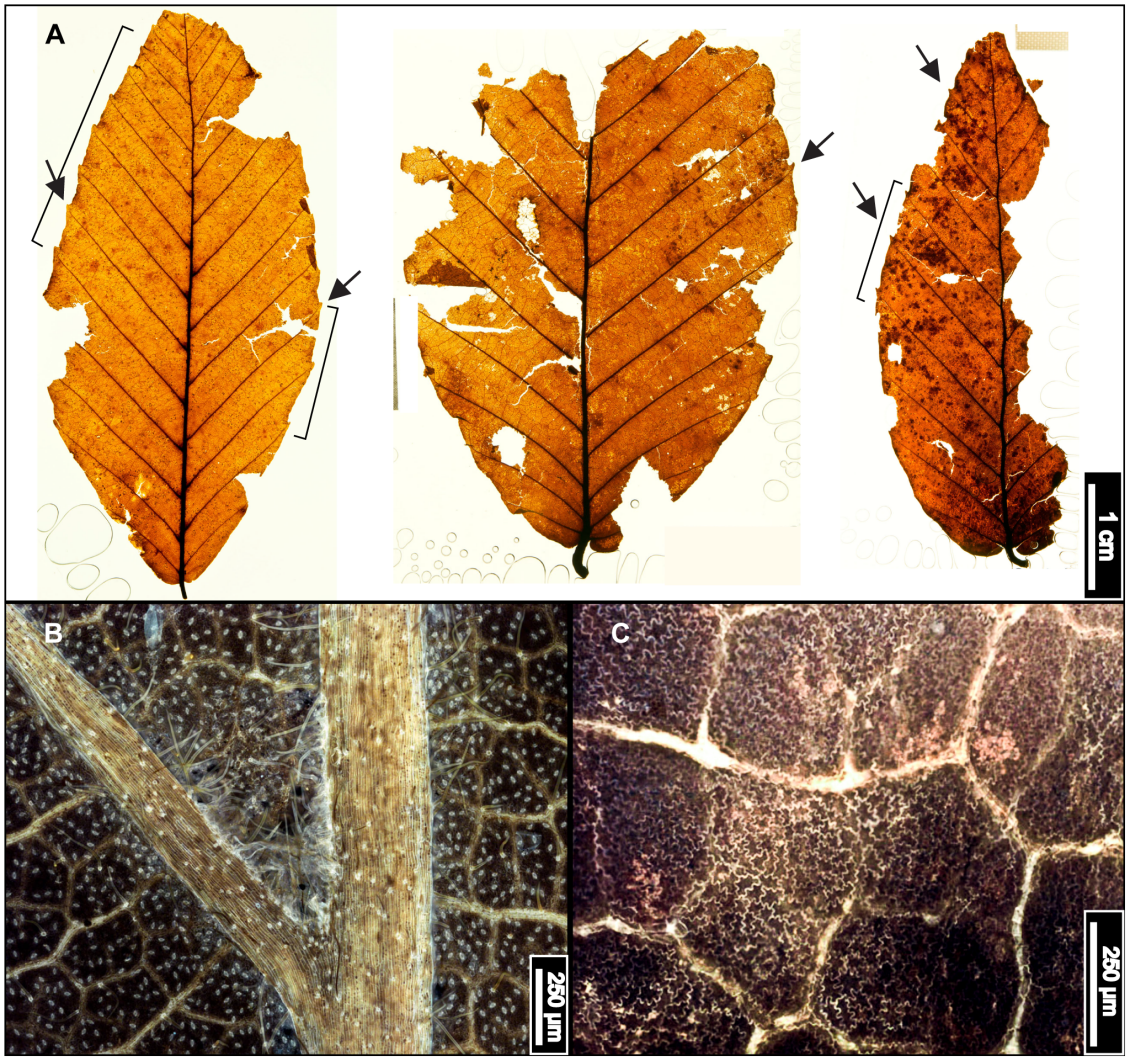
*Fagus grandifolia* (American Beech). (A) Whole subfossils from left to right: EMS 419556, EMS 419558, and EMS 419560, with regularly spaced, straight, parallel secondary veins, each ending in one rounded tooth. Arrows indicate well-preserved teeth; brackets signify extent of preserved margin exhibiting these features. Specimens also exhibit short petioles and convex (EMS 419556) and cordate (EMS 419558, EMS 419560) base shapes. (B) Epifluorescence image shows dense solitary and filiform axillary trichomes, as well as a puberulent cuticle surface (EMS 419556). (C) Highly buttressed adaxial epidermal cells (EMS 419556). Epifluorescence image.

EMS 419556– EMS 419577 (Fig. 9).

#### Relevant distinguishing features

*Fagus grandifolia* (American Beech) leaves are most notably characterized by straight, regularly spaced, parallel secondary veins, each ending in a single, sharp tooth. The cuticle surface is puberulent between sericeous veins, and petioles are usually short. Trichomes are mostly unicellular solitary, or multicellular filiform, and occur primarily along the midvein and secondary veins, with the highest density in vein junctions [73]. The apex is typically falcate, and adaxial epidermal cells are highly buttressed [75].

#### Description

Of the 22 *Fagus grandifolia* specimens, the three most complete fossils were scored, ranging from notophyll to microphyll in size. All three laminae are elliptic in shape with medial and basal symmetry and marginal blade attachment. Apices are acute, convex, or falcate. Base shapes range from convex with an acute angle, to cordate with an obtuse angle. The laminar surface is pubescent with surficial glands. The margins are serrate, with regular tooth spacing occurring once per secondary vein and 2–3 teeth/cm. Sinuses are generally rounded, and common tooth shapes are concave/convex, concave/retroflexed, flexuous/convex, straight/convex, and convex/convex. Principal veins are present and terminate at tooth apices; accessory veins are convex. Primary venation is pinnate with 1–3 basal veins. Major secondary veins are craspedodromous, with uniform vein angles. Secondary vein attachment is mostly excurrent. Major secondary spacing is regular but can decrease proximally. Intercostal tertiary veins are highly variable, ranging from sinuous, straight, or convex opposite percurrent, to alternate percurrent, and sometimes irregular reticulate; they are consistently obtuse to the midvein, with a proximally increasing vein angle. Epimedial tertiaries are mixed, exhibiting opposite and alternate percurrent as well as reticulated fabric. Proximal course is either perpendicular or slightly obtuse to the midvein, while the distal course is parallel to the intercostal tertiary veins. Exterior tertiary vein course is variable. Quaternary vein fabric is irregular reticulate, and quinternary vein fabric is regular reticulate. Areolation exhibits good development, and marginal ultimate venation is either looped or terminates at the margin. Costal veins are pubescent with solitary and filiform trichomes, especially in vein junctions.

#### Remarks

The fossils appear similar to other northeastern species of Fagaceae, including *Quercus muehlenbergii (*Chinkapin Oak) and *Castanea dentata* (American Chestnut), but they are distinguishable using tooth shape and trichome types. The leaf surface of *Q. muehlenbergii* generally lacks the long solitary trichomes seen in *F. grandifolia,* in favor of short, tufted fasciculate and multiradiate trichomes [73]. *Quercus muehlenbergii and C. dentata* also have acute, incurved teeth versus the sharp, but comparatively more rounded, teeth of *F. grandifolia* [81]. The fossils exhibit straight, parallel, secondary veins, each ending in a single rounded tooth (Fig. 9A), short petioles with cordate or convex bases (Fig. 9A), dense trichomes in vein junctions (Fig. 9B), and buttressed adaxial epidermal cells (Fig. 9C). The subfossils were therefore classified as *Fagus grandifolia.*

This species is native to the northeastern United States, ranging from southern Ontario and Michigan to northern Florida, and extending west to Texas and Oklahoma. Isolated, high-altitude populations of American Beech have also been found in New Mexico and Utah [78], [80], but in the northeastern USA, *F. grandifolia* generally grows at lower altitudes [87]. The American Beech is a large tree, growing to 20–24 m in height, with a rounded crown composed of many spreading horizontal branches [80]. *Fagus grandifolia* inhabits moist, rich soils of uplands, or well-drained lowlands [80], [81], [87], and it sometimes forms either dense patches or larger, pure stands due to vegetative propagation from stumps and trunks of young trees [80], [81]. The American Beech is considered a facultative upland species [78].

GENUS *Quercus* L.

SPECIES *Quercus* cf. *Q. alba* L.

### Referred Material

**Figure 10:**
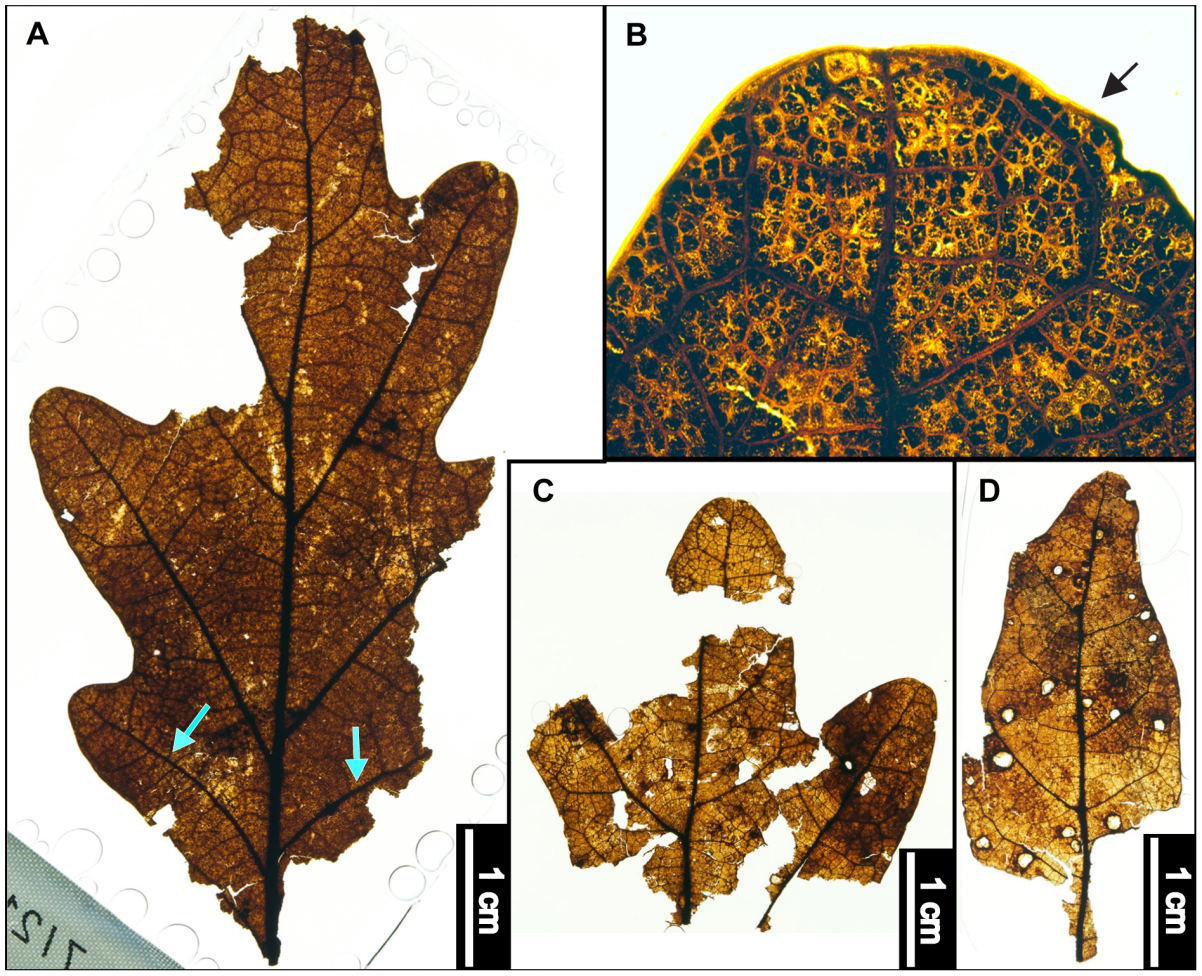
*Quercus* cf. *Q. alba* (Eastern White Oak). (A) Whole White Oak subfossil EMS 419510, with arched secondary veins (arrow). (B) Lobe of EMS 419510, with retuse apex and fimbrial vein (arrow). (C) EMS 419508, showing “triple crown” feature of a true leaf apex. (D) EMS 419509, showing variation in lobe morphology and insect damage.

EMS 419508– EMS 419515 (Fig. 10).

#### Relevant distinguishing features

*Quercus alba* (White Oak) leaves are elliptic, with a decurrent, cuneate base [85], (often) a triple-lobed apex, and entire margins composed of 5–11 rounded lobes, each ending in a retuse, asymmetrical apex [81], [83]. Lobes have moderate to deep, rounded sinuses [84]. A fimbrial vein is present along the margin. Lobes have moderate to deep, rounded sinuses [84]. *Quercus alba* leaves are essentially glabrous [85], although this species sometimes has simple or fasciculate trichomes [72]. Leaf surfaces also contain a high density of randomly oriented stomata whose guard cells form evident T- junctions [75].

#### Description

The largest *Quercus* cf. *Q. alba* fossil specimen was scored because it exhibited the most preserved features; all other samples were fragments. The lamina is microphyll in size, pinnately and moderately lobed, and elliptic in shape with medial symmetry, an entire margin, and a smooth, glabrous surface texture. The distalmost portion of the terminal apex was not preserved, although all other lobes on the specimen end in retuse apices. Primary venation is pinnate. Major secondaries are craspedodromous and arched, with irregular spacing, inconsistent vein angles, and decurrent attachment to the midvein near the leaf base. A fimbrial vein is present on all samples with preserved margins. Intersecondary veins are present, usually within the distal portion of the leaf. Proximal and distal intersecondary courses are parallel to major secondaries. Intersecondary lengths are <50% of subjacent secondary veins and occur at <1 per intercostal area. Intercostal tertiaries are straight and convex opposite percurrent, with some alternate percurrent veins. They are obtuse to the midvein, and vein angle increases exmedially. Epimedial tertiaries are opposite percurrent, with a proximal course perpendicular to the midvein and a distal course parallel to the intercostal tertiaries. Most exterior tertiary veins are looped, although some terminate at the margin. Quaternary vein fabric is mixed percurrent to irregular reticulate, and quinternary vein fabric is consistently irregular reticulate. Sixth order veins are sometimes random reticulate, but they mostly end in unbranched or singly branched freely ending veinlets. Areolation is good to moderate.

#### Remarks

Regional species within the White Oak Group (*Quercus* subgenus *Quercus*) generally have rounded lobe apices, differentiating them from the acute, bristle-tipped lobes of the Red Oak Group (*Quercus* section *Lobatae*). The only white oak species native to the region that lacks these characteristics is *Quercus muehlenbergii* (Chinkapin Oak), which has a serrate margin with regularly spaced, curved teeth [81], [84], unlike the subfossils. The leaf surfaces of the *Quercus* cf. *Q. alba* fossils from DM are apparently glabrous, which is a distinguishing characteristic of *Q. alba* but could also be taphonomic. Additionally, the subfossils exhibit other characteristics of *Q. alba*, including arched secondary veins (Fig. 10A), rounded lobes with retuse, asymmetrical apices (Fig. 10A–C), a strong fimbrial vein (Fig. 10B), and triple-lobed apices (Fig. 10C), and were therefore tentatively classified as such. We note that a fragmentary cupule and attached nut of an acorn were found (Table S1) but could not be further identified.

The eastern White Oak is native to North America, ranging from southern Ontario and Quebec, east to Maine, south to northern Florida, and west to Minnesota and Texas [78]. *Quercus alba* usually grows to 24–30 m, or taller, in height, and it forms a large rounded crown with wide-spreading, stout, horizontal branches [80]. The White Oak prefers moist, rich upland soils, often on ridges or slopes, or well-drained lowlands [84], and tends to form pure stands [80]. This species is classified as facultative upland [78], and it is possibly the most abundant native hardwood tree species in North America [84].

GENUS *Quercus* L.

SECTION *Quercus* section *Lobatae* Loudon.

### Referred Material

**Figure 11:**
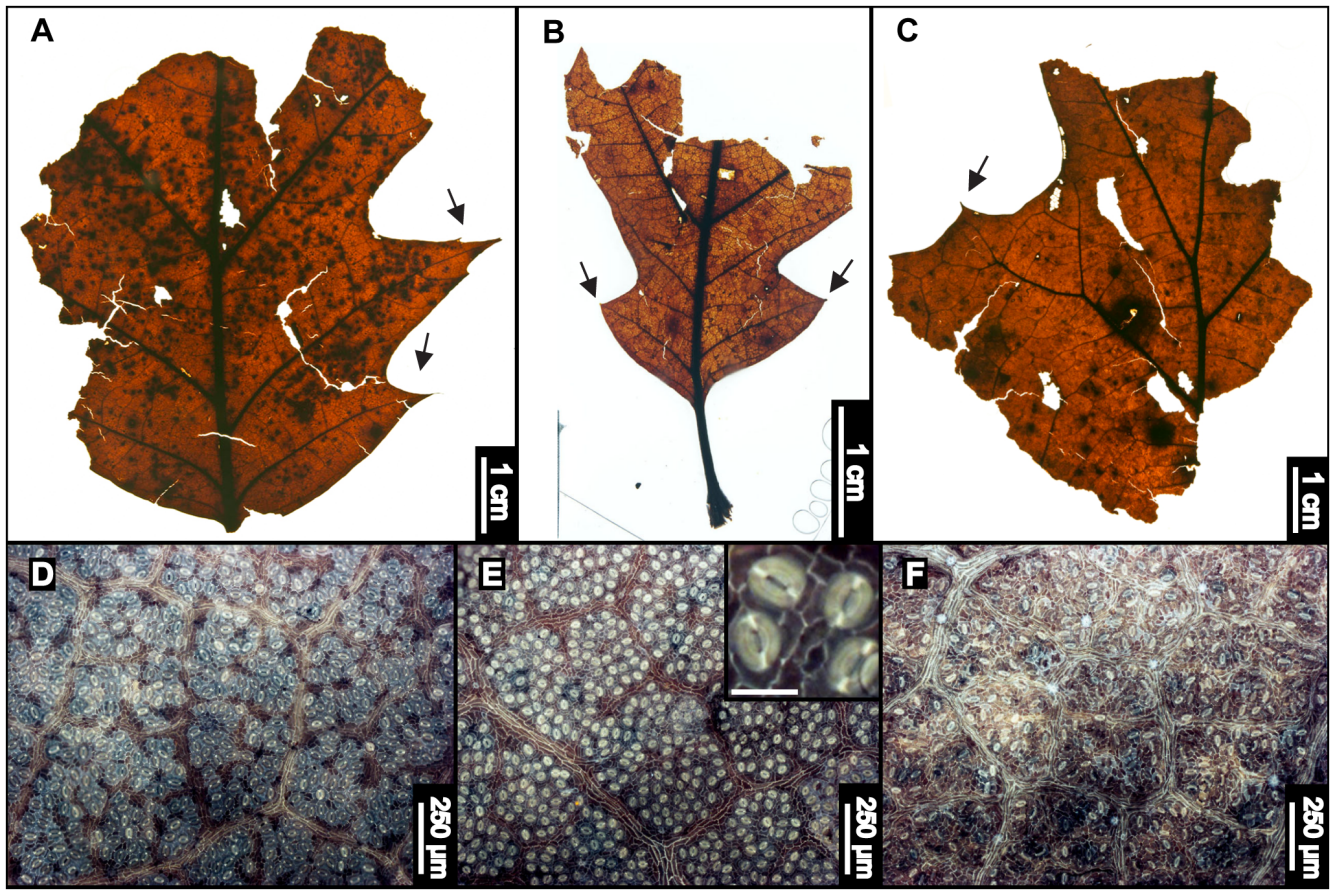
*Quercus* section *Lobatae* morphotypes (Red Oak subgroup). (A) EMS419516. (B) EMS419524, juvenile. (C) EMS419523, section of what was a very large leaf with preserved secondary veins, but no preserved midvein. Arrows point to preserved acute, bristle-tipped lobes. (D–F) Examples of cuticle variation among specimens. Inset in middle image shows distinctive T-shaped guard cell junctions; inset scale bar = 30 microns. Epifluorescence images.

EMS 419516– EMS 419554 (Fig. 11).

#### Relevant distinguishing features

Unlike the White Oak Group, the majority of *Quercus* section *Lobatae* (red oaks) are characterized by elliptical leaves with oblong, acute, asymmetrical lobes, sometimes ending in irregular bristle tips. These lobes can be distally expanded and are separated by shallow or deep sinuses. Red oak margins also contain a fimbrial vein. Although there are species belonging to the red oak group with entire margins, these still possess at least a few marginal bristles. Leaf surfaces also contain a high density of randomly oriented stomata whose guard cells form evident T- junctions [75].

#### Description

All four scored subfossils lack apices and range from microphyll to notophyll in estimated size. Because apices were not preserved, laminar shapes could not be precisely determined, but all could be either elliptic or oblong. The laminae are also pinnately and moderately to deeply lobed. Specimens exhibit medial symmetry and a marginal, asymmetrical, basal insertion onto the petiole. Margins are entire except for bristle-tips at lobe apices. Sinuses are usually angular and somewhat v-shaped but can also be rounded. Base angles are obtuse, and base shape can be rounded, slightly cordate, or asymmetrically decurrent. Primary venation is pinnate. Major secondaries are craspedodromous, arched, and have basally decurrent attachment. Secondary vein spacing is roughly regular but sometimes slightly decreases proximally, and vein angles are either uniform or smoothly decrease proximally. A fimbrial vein is present in all specimens with preserved margins. Intersecondaries are <50% of subjacent secondaries and occur at <1 per intercostal area. Intersecondary proximal courses are perpendicular to the midvein or parallel to the major secondaries, and distal courses are either parallel to the subjacent major secondary or reticulate. Intercostal tertiary veins are either straight or sinuous opposite percurrent, or are alternate percurrent. They are obtuse to the midvein and increase in vein angle proximally. Epimedial tertiaries range from mixed percurrent to reticulate, having a proximal course that is either perpendicular or obtuse to the midvein and a basiflexed distal course. Exterior tertiary veins are variable. Quaternary vein fabric is alternate percurrent or regular reticulate. Quinternary vein fabric is regular reticulate. Sixth order veins are regular reticulate or end in simple, freely ending veinlets, which are mostly singly branched but can also be unbranched or dichotomous. Areolation exhibits good development. Cuticle surface texture is essentially smooth, with scattered laminar glands and no observable trichomes.

#### Remarks

Identifying the subfossil red oak specimens to species level based on leaves alone was not feasible for multiple reasons, including the substantial amount of interspecies morphological similarity, the ample amount of intraspecies morphological variation, and the tendency of species within this section to hybridize [81]. A complete lack of observable trichomes on all red oak morphotype specimens further impeded species identifications. The subfossils are distinguishable as members of the Red Oak Group due to their acute, bristle-tipped lobes (Fig. 11A–C).

Taxa belonging to the Red Oak Group vary widely in their environmental preferences [78], [81], and based on the habitat ranges of the other taxa identified within the subfossil assemblage, the Red Oak Group morphotypes could represent a number of possible species, excluding those without acute-lobed morphology. Some of the most likely species represented could be *Quercus shumardii* (Shumard Oak) and *Q. palustris* (Pin Oak), which are facultative to facultative wetland taxa that are associated with a variety of riverine and wetland habitats [78], [81], [88], or *Q. rubra* (Red Oak) and *Q. coccinea* (Scarlet Oak), which prefer dry to moist woodlands on upland ridges and slopes [80], [81], [89], [90] but also grow on higher spots along valley bottom margins at wetland sites. It is also possible that purely upland species such as *Q. velutina* (Black Oak) and *Q. ilicifolia* (Bear Oak) are present [78]. Besides *Q. ilicifolia*, which is a shrub that grows up to 6 m in height [80], all species within the Red Oak subgroup are large trees, typically growing to 20–30 m in height [81].

ORDER Salicales.

FAMILY Salicaceae.

GENUS *Salix* L.

SPECIES *Salix* sp.

### Referred Material

**Figure 12:**
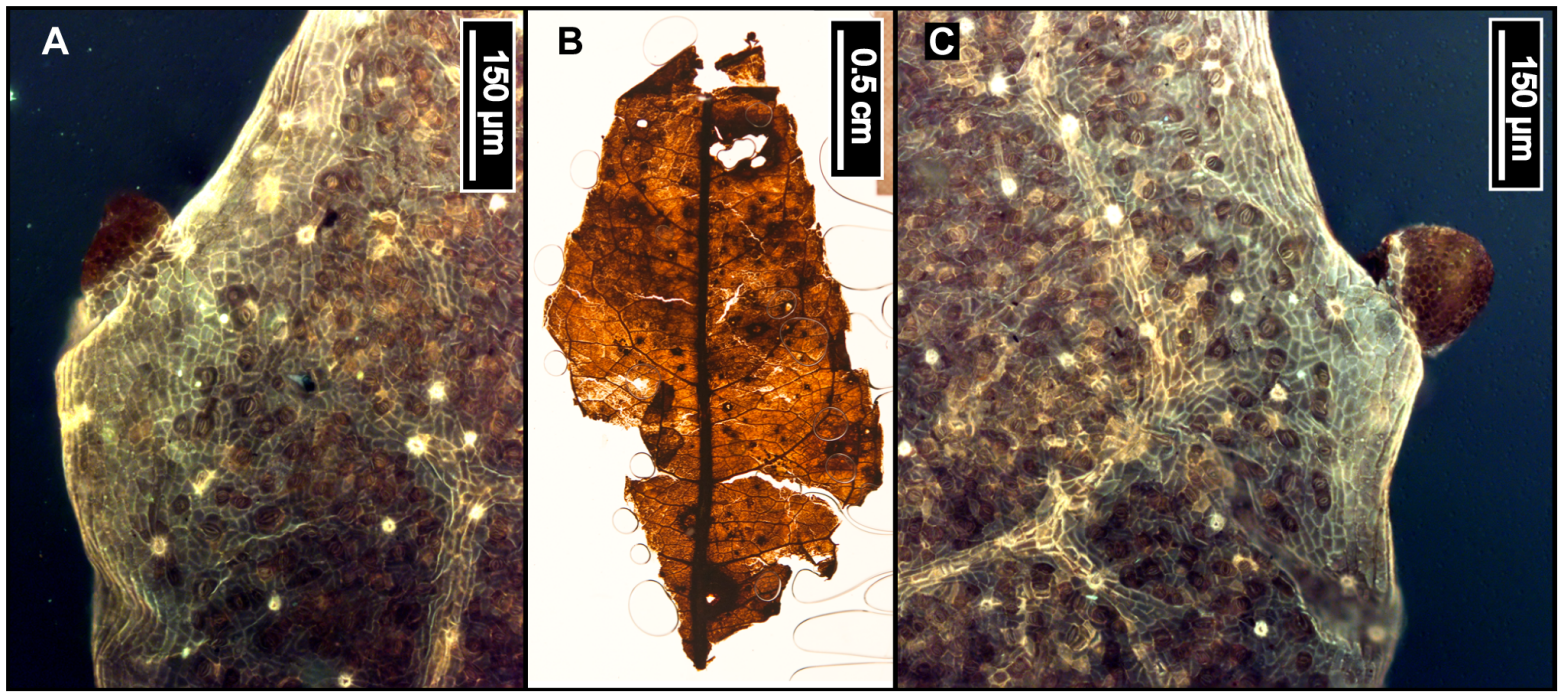
*Salix* sp. (Willow). (A) Characteristic salicoid tooth from left margin (as viewed) of whole subfossil. Epifluorescence image. (B) Complete specimen EMS 419501. (C) Well-defined salicoid tooth from right margin (as viewed) of subfossil, also showing well preserved stomata. Epifluorescence image.

EMS 419501 (Fig. 12).

#### Relevant distinguishing features

Most *Salix* spp. (willows) are shrubs or small trees that grow in dense thickets along streams and in other wet areas [84]. Leaf surfaces of this genus are generally pubescent. The laminae are at least two times longer than they are wide and have characteristic salicoid teeth, with dark, round setae attached to teeth apices [81], [91].

#### Description

The single subfossil fragment is a distal portion of an apex and is medially symmetrical. The laminar margin is minimally serrate, with rounded sinuses. Teeth are salicoid, with spherulate glands at the apices. They are regularly spaced, with approximately 5 teeth/cm. Tooth principal veins are present and terminate at the tooth apex. The leaf apex angle is acute. Primary venation is pinnate. Major secondary veins are simple brochidodromous, with irregular spacing. Vein angles smoothly increase proximally. Intersecondary veins are weak and grade into epimedial tertiary veins; they are half the length of the subjacent secondary, or longer, and occur at <1 per intercostal area. Proximally, they are parallel to the major secondaries, and distally they dichotomize to join adjacent secondary veins. Intercostal tertiary veins are either convex to sinuous opposite percurrent or irregular reticulate. They are consistently obtuse to the midvein. Epimedial tertiaries are mixed percurrent. Proximally, they are either perpendicular or acute to the midvein, and distally they are parallel to the intercostal tertiaries. The majority of exterior epimedial tertiaries are looped, although some terminate at the margin. Quaternary vein fabric is mixed percurrent, and quinternary vein fabric is freely ramifying. Freely ending veinlets are mostly one-branched, with simple terminations. Marginal ultimate venation is looped. Areolation exhibits poor development. The laminar surface is rugose due to high vein relief and is apparently glabrous, possibly due to taphonomic processes.

#### Remarks

Identifying willows to species level based on isolated leaves proves difficult because branching patterns, leaf attachment, and catkin morphology are the typical diagnostic characters, and hybridization is common [92]. Only two genera within family Salicaceae, *Salix* and *Populus,* are native to the region [81], and both typically have salicoid teeth. *Populus* leaves can be distinguished from those of *Salix* because they are about as wide as they are long [81]. The morphotype presented here, though fragmentary, is clearly much longer than it is wide and is assigned to *Salix* (Fig. 12B). *Salix* is found in temperate to high latitude areas worldwide but primarily in the northern hemisphere, with ca. 100 species native to North America [81]. Of the species that are native to Pennsylvania, all except one (*Salix humilis*, Prairie Willow) are either wetland obligate or wetland facultative species [78]. *Salix humilis* has an entire margin and is frequently tomentose on the abaxial leaf surface [81], [92]. The *Salix* subfossil described here does not have these characteristics, and it is therefore considered to represent a willow species with wetland obligate or wetland facultative affiliation.

ORDER Sapindales.

FAMILY Aceraceae.

GENUS *Acer* L.

SPECIES *Acer spicatum* Lam.

### Referred Material

**Figure 13:**
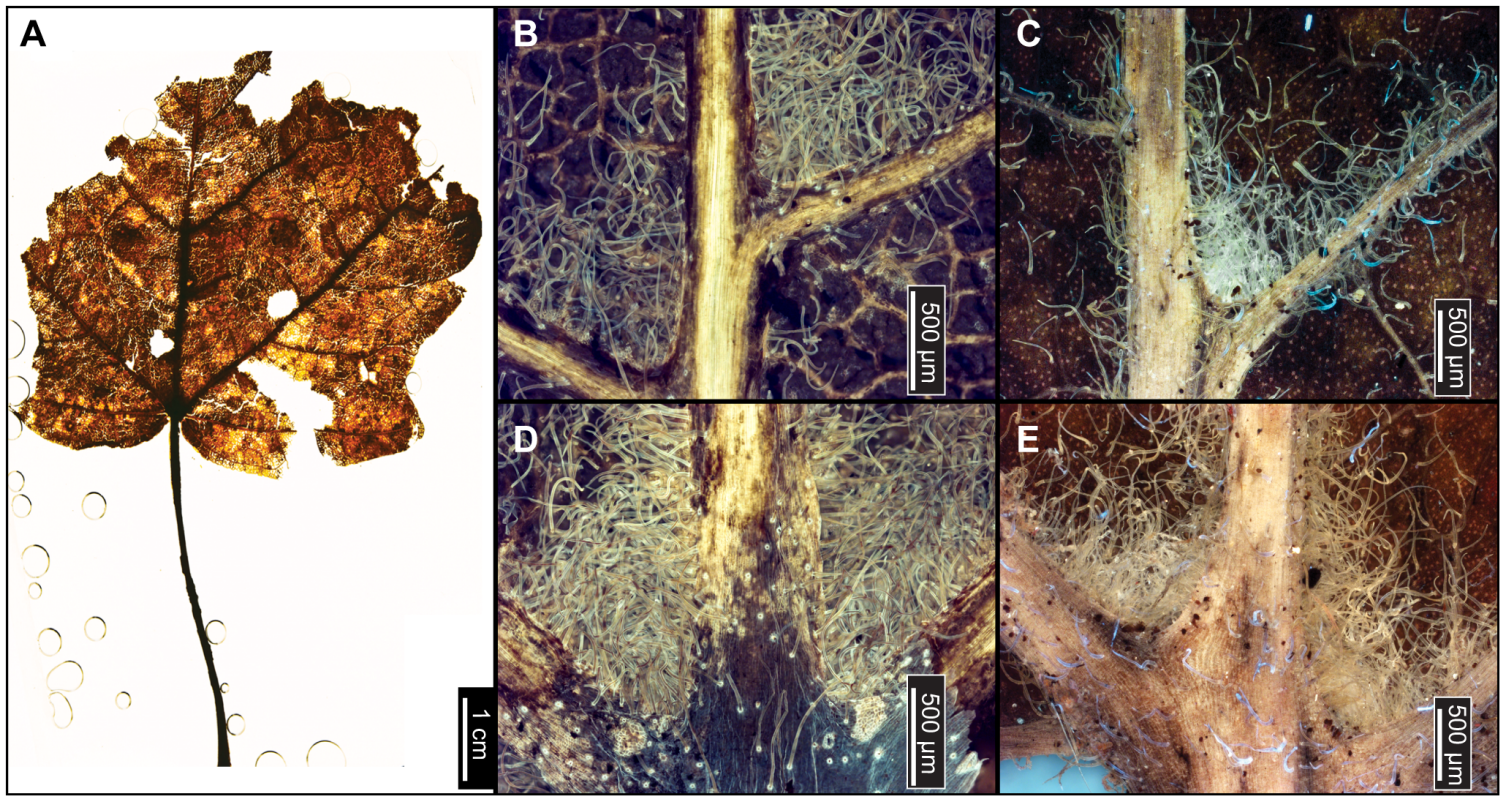
*Acer spicatum* (Mountain Maple). (A) Whole subfossil EMS 419502. (B) EMS 419502, axillary tufts of acicular, aduncate, and filiform trichomes. Epifluorescence image. (C) Dense acicular, aduncate, and filiform basal trichomes of sample EMS 419502. Epifluorescence image. (D) and (E) *Acer spicatum* reference image from sample M5-2 of the Allegheny National Forest, Pennsylvania collection [72]. Epifluorescence images. (D) Image shows axillary tufts with the same types and configuration of trichomes as (B), while (E) exhibits the same dense basal trichomes seen in (C).

EMS 419502 (Fig. 13).

#### Relevant distinguishing features

Leaves of *Acer spicatum* (Mountain Maple) are broadly ovate or orbicular, with 3–5 short, broad lobes forming v-shaped sinuses, and a coarsely serrated margin [80], [82], [83]. Leaves of *A. spicatum* exhibit palmate venation, with 5–7 primary veins. The Mountain Maple is pubescent on the petiole and on the abaxial leaf surface, which both exhibit scattered acicular and solitary trichomes. The vein junctions and petiole attachment area are densely villous, with acicular, filiform, and aduncate trichomes [80], [82].

#### Description

The subfossil lamina is microphyll in size, orbicular in shape, and palmately lobed. The preserved petiole portion is 3 cm long and pubescent, with a marginal blade attachment. The apex and true margin are not preserved. Primary venation is basal actinodromous, with five distinctive primary veins. Base shape is lobate, with a reflexed base angle. Surface texture is highly pubescent, especially at vein junctions. Major secondary veins are excurrent. Interior secondaries are present but poorly preserved. Intercostal tertiary veins are obtuse to the midvein, are either alternate percurrent or sinuous opposite percurrent, and increase in angle exmedially. Epimedial tertiaries are reticulate. Both quaternary and quinternary vein fabrics are irregular reticulate. Areolation shows good development.

#### Remarks

*Acer spicatum* leaves are morphologically similar to other species of maple with 3–5 broad lobes, particularly *Acer pensylvanicum* (Striped Maple). The Striped Maple can be distinguished from the Mountain Maple because it has a considerably more finely serrated margin and because it is densely tomentose over the whole abaxial surface [80]. Additionally, leaves of the Striped Maple tend to have pointed lobes and only three basal veins [83]. The single subfossil has five distinctive primary veins (Fig. 13A), a pubescent abaxial surface with scattered trichomes, a pubescent petiole, and dense, villous vein junctions (Fig. 13B, D). Even though the true margin of the subfossil was not preserved, it is classified as *Acer spicatum* based on the combination of these other distinguishing features.

*Acer spicatum* is native to northeastern North America and ranges along the eastern half of the continent, from Saskatchewan and Newfoundland to the southern Appalachians through Georgia and Alabama [78], [80]. The Mountain Maple is a small tree or shrub usually growing no larger than 7–8 m in height, with a crown of slender, upright branches [82]. This species prefers moist rocky uplands, especially on mountains, and is a common understory tree of hardwood forests [80]; it is classified as facultative upland [78].

GENUS *Acer.*

SPECIES *Acer rubrum* L.

### Referred Material

**Figure 14:**
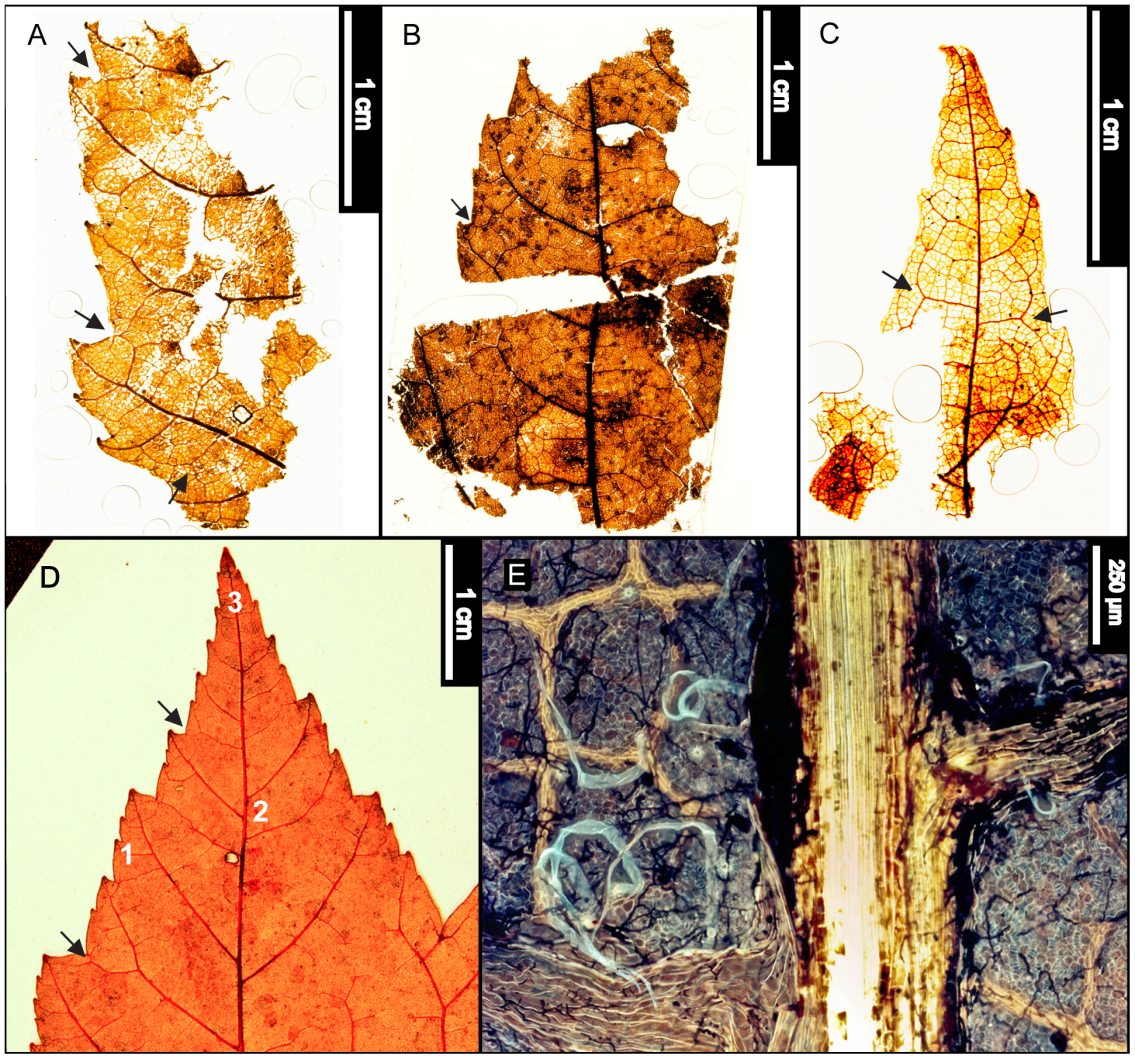
*Acer rubrum* (Red Maple). (A) Coarse, compound serrated margin of subfossil EMS 419504. (B) Middle portion of a lobe, subfossil EMS 419505. (C) Lobe apex, subfossil EMS 419503. (D) *Acer rubrum* reference image from sample M9-1 of the Allegheny National Forest, Pennsylvania collection [76]. Numbers correspond to the probable areas of a lobe represented by EMS 419504, EMS 419505, and EMS 419503. Arrows in (A–C) point to dichotomizing tertiary veins near the margins. (E) Epifluorescence image showing puberulent vein junctions with aduncate trichomes and stomatal configuration on EMS 419505.

EMS 419503– EMS 419505 (Fig. 14).

#### Relevant distinguishing features

Leaves of *Acer rubrum* (Red or Swamp Maple) are broadly ovate with 3–5 lobes, and they have a compound serrated margin composed of coarse and irregular teeth. Leaves typically have five primary veins [80], as well as a perimarginal vein, and dichotomizing minor secondary and tertiary veins near the margin. Leaves are glabrous to puberulent on the abaxial surface and on costal veins, with aduncate trichomes in vein junctions [80]–[82].

#### Description

Each of the three subfossils representing *Acer rubrum* preserves various areas of the laminar surface, including a lobe apex approximately 2 cm long, a segment of the mid-leaf margin approximately 3 cm long, and the central portion of a lobe. Combining the characteristics of all specimens, the apex is straight and acute, and the leaf surface is generally smooth with a serrated margin. Tooth spacing is irregular, with one or two orders of teeth, and 3–4 teeth/cm. Depending on tooth shape and crowding, the sinuses are mostly angular but can be rounded. Principal tooth shapes include concave/convex, concave/straight, concave/retroflexed, flexuous/retroflexed, and flexuous/concave. Principal veins terminate in tooth apices. Accessory veins are usually convex but can rarely be concave, depending on the size and shape of the tooth. Primary venation is not preserved. Agrophic veins are present, and minor secondary veins are craspedodromous to simple brochidodromous. Major secondary veins are craspedodromous with excurrent attachment, but accurate spacing could not be determined from the fragments. Variation in secondary vein angle could be either uniform or smoothly increasing proximally. Intersecondary vein lengths are >50% of subjacent secondaries and occur at ∼1 (2) per intercostal area. Proximal course is either parallel with the major secondaries or perpendicular to the midvein, and the distal course is either perpendicular to the subjacent major secondary or reticulate. Intercostal tertiary veins are obtuse to the midvein and can be mixed percurrent, straight or sinuous opposite percurrent, or alternate percurrent. Epimedial tertiaries are either alternate percurrent or reticulate. Exterior tertiary course is looped. Quaternary vein fabric is usually regular reticulate but can be irregular. Quinternary veins freely ramify. Areolation development is moderate to good. Freely ending veinlets are mostly unbranched, although some have one branch with simple termination. Marginal ultimate venation is looped. The preservation quality of these three specimens was low, and the majority of the cuticle was lost. A few remaining trichomes were found on one specimen, within vein junctions (Fig. 14E).

#### Remarks

*Acer rubrum* leaves are morphologically similar to several other species of maple, including *Acer saccharum* (Sugar Maple) and the closely related *Acer nigrum* (Black Maple). Both these species also have 3–5 palmate lobes and coarsely serrated margins, but they usually have deeper, more pointed lobes than the Red Maple [80], [81]. These species can also be differentiated based on trichome configurations. Unlike the Red Maple, which is glabrous to somewhat puberulent, the Black Maple and the Sugar Maple are commonly sericeous, with long trichomes on or near veins [81], [84]. The subfossils exhibit coarse, compound serrated margins (Fig. 14A–C) and dichotomizing exterior tertiary veins that rejoin secondary veins instead of terminating at the margin. These features are typical of Red Maple leaves (most clearly seen in Fig. 14C). When preserved, they also have puberulent vein junctions with aduncate trichomes (Fig. 14E). Therefore, these subfossils were classified as *Acer rubrum*.

Red Maple is native to North America and is widespread in eastern Canada and the United States west to Texas, Oklahoma, and Minnesota, and south to the tip of Florida [78], [80]. Red Maple becomes a tall tree, ranging from 24–27 m in height, and forms a narrow or rounded crown [80], [82]. This species is characterized as obligate or facultative wetland [78], but it is adapted to a wide array of climatic and environmental conditions. In Pennsylvania, *Acer rubrum* is commonly associated with moist or wet soils of stream banks, swamps, and valleys, but it can also grow on upland slopes and dry ridges in mixed hardwood forests [80], [81].

ORDER Scrophulariales.

FAMILY Oleaceae.

GENUS *Fraxinus* L.

SPECIES *Fraxinus nigra* Marsh.

### Referred Material

**Figure 15:**
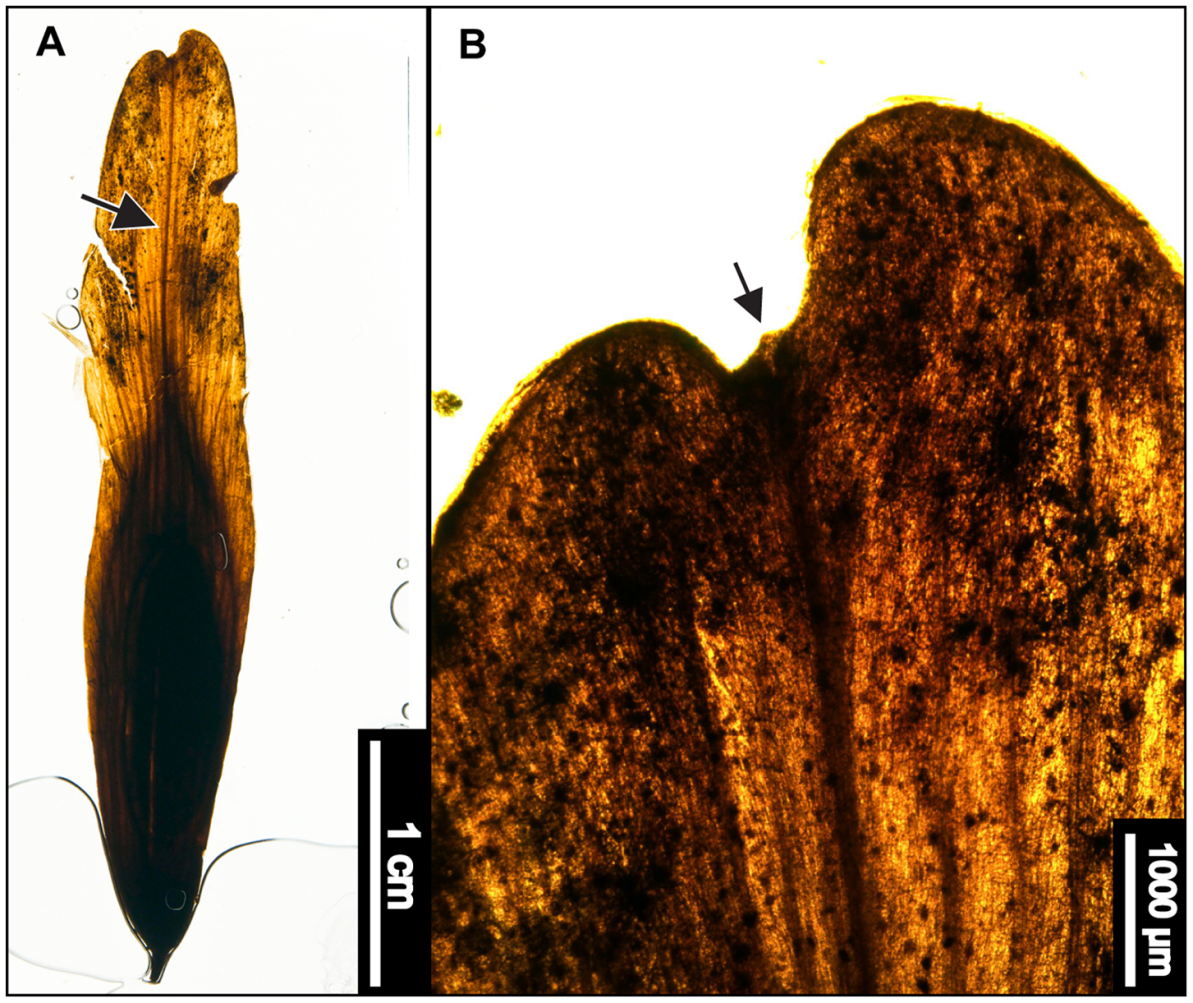
*Fraxinus nigra* (Black Ash) samara. (A) Whole subfossil EMS 419507, showing retuse apex, the flat, elliptical seed body with the wing extended to the base, and the midvein (arrow). (B) Close up of midvein and retuse, notched apex with small projection (arrow).

EMS 419507 (Fig. 15).

#### Relevant distinguishing features

*Fraxinus nigra* (Black Ash) is the only species recovered in this study that is solely represented by its fruit. Black Ash samaras are typically 2.5–4 cm in length and contain a flat-bodied seed located in an almost-invisible seed cavity that is barely thicker than the wing itself. Wings of *F. nigra* fruits are broad, oblong, and extend to the base of the seed cavity, although they may be very closely adpressed to the seed toward the base. A midvein runs down the entire length of the wing, connecting the seed body to the wing apex. The samaras also have retuse, notched apices and can develop a twisted shape [80], [83].

#### Description

Short pedicel or pedicel fragment with samara of length 3.8 cm, width 0.67 cm. Seed flattened, ovoid, body length 2.52 cm, width 0.56 cm. Wing elliptic, with a midvein, numerous low-angled secondary veins, and a retuse, notched apex, extending to base of seed though scarcely wider along the seed basal half.

#### Remarks

Fruits of the Black Ash are similar to those of other ash species commonly found in the region, including the Green Ash (*Fraxinus pennsylvanica*) and the White Ash (*Fraxinus americana*), as well as the fruit of the unrelated Tulip Tree (*Liriodendron tulipifera*). Black Ash samaras differ from those of *L. tulipifera* because, even though they can become twisted, they lack the thickened basal ridge and are flat in comparison to the curved wings of the Tulip Tree fruit [82]. Both White and Green Ash samaras are often longer than those of the Black Ash and the fossil, and, unlike *F. nigra* and the fossil, also contain seed bodies that are much thicker than the winged portion of the samara. Additionally, although these attributes can vary, the wing of the Black Ash extends all the way to the base of the seed (Fig. 15A), whereas the White Ash wing extends from a point 1/3 of the way up the length of the seed body [83], and the Green Ash wing begins at or slightly past the middle of the elongated seed. Samaras of the Green Ash are therefore typically cuspate in appearance, compared with the rounded samaras of either the White or Black Ash [83]. Because the subfossil samara is approximately 4 cm long, contains a flattened seed body with a wing that extends to the base of the seed, and has a notched apex (Fig. 15B), it is classified as *Fraxinus nigra*.

*Fraxinus nigra* is native to North America, extending from Manitoba and Newfoundland south to Virginia and Iowa [78], [82]. It typically grows 9–15 m tall, sometimes up to 25 m [81], and forms a narrow, rounded crown of upright branches [80]. *Fraxinus nigra* occurs in both coniferous and hardwood forests, preferring wet soils near streams, swamps, and peat bogs [80], and is classified as a facultative wetland species [78].

ORDER Hamamelidales.

FAMILY Platanaceae.

GENUS *Platanus* L.

SPECIES *Platanus occidentalis* L.

### Referred Material

**Figure 16:**
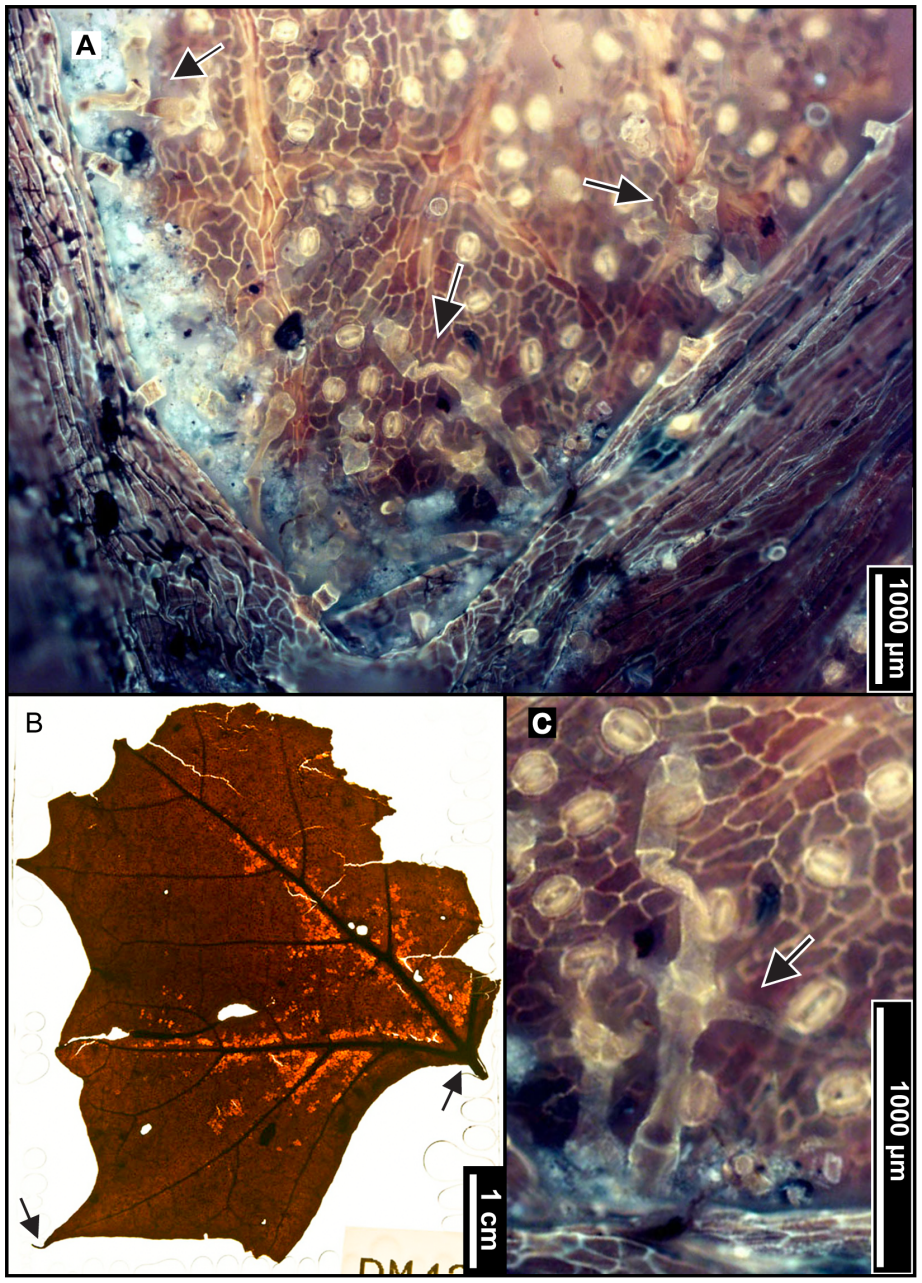
*Platanus occidentalis* (American Sycamore). (A) Arrows point to characteristic multiradiate (tall and branched) trichomes in vein junctions of subfossil EMS 419506. Epifluorescence image. (B) Whole subfossil EMS 419506, showing a bristle-tipped lobe. (C) Close-up of preserved multiradiate trichome in vein junction from image (A).

EMS 419506 (Fig. 16).

#### Relevant distinguishing features

Leaves of *Platanus occidentalis* (American Sycamore) are highly variable, but they are typically very large and broadly ovate in shape [84]. They have palmate venation with 3–5 palinactinodromous primary veins. American Sycamore leaves also have 3–5 lobes that are often wider than they are long, forming shallow concave sinuses [86]. The leaves can have either entire or coarsely toothed margins, sometimes with short awn (bristle) tips on acuminate tooth apices [80], [83], [86]. Veins are persistently tomentose on the abaxial surface [86], exhibiting multiradiate (candelabraform) trichomes with whorls of arms in vein junctions [93].

#### Description

The single subfossil is palmately lobed with an entire margin, except for one preserved bristle-tipped lobe, and a marginal blade attachment. The apex is not preserved, and the base is lobate with a reflexed angle. The surface texture is pubescent, with scattered surficial glands. Primary venation is basal actinodromous with three basal veins. Compound agrophic veins are present. Both major and minor secondary veins are semicraspedodromous. Intercostal tertiary veins are straight or sinuous opposite percurrent, are obtuse to the midvein, and increase in angle exmedially. Epimedial tertiaries are mixed percurrent, with a proximal course perpendicular to the midvein and a basiflexed distal course. Exterior tertiary veins terminate at the margin. Quaternary vein fabric is mixed percurrent. Quinternary vein fabric is irregular reticulate. Freely ending veinlets are absent, and areolation shows good development. Some preserved, large candelabraform trichomes are evident in vein junctions along the midvein.

#### Remarks

The American sycamore is similar to the hybrid London Planetree (*Platanus* × *acerifolia*) and the Sycamore Maple (*Acer pseudoplatanus*), native to Europe and Western Asia [84], respectively, that are widely cultivated in Pennsylvania today. The fossil leaf does not closely resemble any other native species that would have been present at the time of deposition. The classification of the subfossil as the American Sycamore is further reinforced by characteristic multiradiate (candelabraform) trichomes in vein junctions (Fig. 16A, C).

This species is native to the northeastern U.S. and extends from Ontario and southwestern Maine to Florida, and west to central Texas and Nebraska [78]. Isolated populations also occur in northeastern Mexico [80]. The American Sycamore grows between 18 and 30 m tall, sometimes up to 50 m [85]. It has the largest trunk diameter of any native hardwood species in Pennsylvania and crooked branches that spread to form a broad, open crown [80]. This species prefers wet soils of streambanks and floodplains as well as the edges of lakes and swamps [84], and it is commonly dominant in mixed forests [80]. The American Sycamore is classified as a wetland or facultative wetland species [78].

## Discussion

The pre-settlement flora recovered from Denlingers Mill consists of eleven identified woody species and morphotypes. It was expected that the majority of species would have facultative or facultative upland affiliations because of local topography around the fossil site, but the presence of lowland trees and unidentified herbaceous remains, most of which appear to be of monocots, reinforces the notion that this leaf mat deposit samples several environments in the transition from valley bottom wetland to true upland ecosystems.

With the exception of the purely upland *Castanea dentata* (American Chestnut), represented by a single leaf fragment, all the other identified woody taxa are associated with wetlands today at least part of the time. Within the subfossil assemblage, the species with the highest relative abundances are *Fagus grandifolia* (American Beech), *Quercus* section *Lobatae* (Red Oaks), and *Betula lenta* (Sweet Birch), all of which have facultative upland associations throughout the northeast. Other facultative upland elements include *Acer spicatum* (Mountain Maple) and *Ostrya virginiana* (Eastern Hophornbeam). The remaining four subfossil species, *Platanus occidentalis* (American Sycamore), *Acer rubrum* (Red Maple), *Fraxinus nigra* (Black Ash), and *Salix* sp. (willow), are commonly either facultative or facultative wetland, and are interpreted as representing the fringing valley-margin forest community between the low-lying wetlands and the facultative upland assemblage just described.

The Denlingers Mill fossil site is unusual in that it preserves both a subfossil and an extant upland slope community from the same, small source area. Even though there are species shared between the subfossil assemblage and modern community, the dominant components of the fossilized old-growth and extant secondary forests vary significantly (see Tables 2, 3, 5). Furthermore, our results illustrate that extreme vegetation shifts, most likely attributable to deforestation and land clearing, have occurred on slopes and other upland areas. These shifts were in addition to, and distinct from, the transformation of the immediately adjacent lowlands, which were mostly affected by milldam construction and accumulation of legacy sediments. However, based on certain species identified both in the modern forest and the subfossil assemblage, such as *Quercus* spp., *Fagus grandifolia*, and *Platanus occidentalis*, it is also possible that some of the hardwood trees around the field site today may be descended from the pre-colonial trees that once inhabited the environs of Denlingers Mill.

**Table 5:** Comparison of fossil floras from Denlingers Mill, White Clay Creek [12], and Big Spring Run [8].

Site	Denlingers Mill	White Clay Creek	Big Spring Run
**Data** **Source**	Leaf macrofossils, fruit macrofossils(*Fraxinus nigra, Ostrya virginiana*)	Leaf macrofossils, fruit macrofossil(*Liriodendron tulipifera*)	Fruit and seed macrofossils
**Location**	West Branch of the Little Conestoga Creek,Lancaster Co., PA	White Clay Creek, Chester Co., PA	Big Spring Run, Lancaster Co., PA
**Age**	113 to 295 Cal BP	217 to 368 Cal BP	140 to 3560 Cal BP
**Herbaceous** **Species**	Remnant herbaceous material recoveredbut none identified.	Remnant herbaceous material recoveredbut none identified.	*Carex stricta, C. crinita, C. stipata, Polygonum* spp., *Eleocharis* spp., *Scirpus* spp., *Najas flexilis*, *Brasenia scherberi*
**Woody** **Species**	*Fagus grandifolia, Quercus* subgenus *Quercus,* *Quercus* section *Lobatae, Betula* spp.,Ostrya virginiana, *Fraxinus nigra, Acer rubrum,* *Acer spicatum, Castanea dentata,* *Platanus occidentalis,* *Salix* sp.	*Acer rubrum, Acer negundo, Alnus serrulata,* *Salix* spp., *Fagus grandifolia, Quercus*subgenus *Quercus, Quercus* section *Lobatae,* *Liriodendron tulipifera*	*Liriodendron tulipifera, Juglans cinerea*
**Community** **Type**	Red Oak-Beech mixed hardwood forest(upslope) and Red Maple-Black Ash forestedswamp (valley margin)	Potential Red Maple-Black Ash forested palustrine swamp (valley margin)	Palustrine wet meadow wetland with herbaceous obligate emergent species and adjacent transitional/upslope communities

### Classification of the Contemporary Forest

Based on the modern stand and leaf litter data (Tables 2, 3; Fig. 4), the contemporary forest surrounding Denlingers Mill gradually shifts from a Red Oak-Sugar Maple forest on the uppermost slopes (ca. 5–8 m above base level), into a northeastern modified successional forest on lower slopes (ca. 2–5 m above base), and a Box Elder floodplain forest nearest to the stream (ca. base −2 m). Riparian buffer habitats characteristically support mixed wetland and facultative forest vegetation, as well as high structural, community, and species diversity, due to variations in substrate, disturbance, and hydrologic regimes. Vegetation in riparian and transitional (slope) environments is driven primarily by stream water levels, often producing a continuum of intermediate forest classification types [94].

In Pennsylvania, the Red Oak-Sugar Maple forest is typically found on moist slopes and is dominated by *Acer saccharum* (Sugar Maple), *Quercus* spp. (red and white oaks), *Liriodendron tulipifera* (Tulip Tree), *Carya* spp. (hickories), and *Nyssa sylvatica* (Black Tupelo). Other constituents include *Betula* spp. (birches), *Fagus grandifolia* (American Beech), *Ostrya virginiana* (Eastern Hophornbeam), and *Carpinus caroliniana* (Eastern Hornbeam) [95]. Its presence as a subfossil suggests that *Castanea dentata* (American Chestnut) was once an important component of this forest type as well, before its near demise from Chestnut Blight [96].

Located between the upper slope Red Oak-Sugar Maple forest and the floodplain Box Elder forest, is a mesic, physiognomically variable northeastern modified successional forest, which is typical of disturbed sites that have been cleared for agriculture or otherwise heavily modified in the past [95]. This forest type includes *Liriodendron tulipifera* (Tulip Tree), *Prunus serotina* (Black Cherry), *Juglans nigra* (Black Walnut), *Ulmus* spp. (elms), *Quercus* spp. (oaks), *Betula* spp. (birches), and various maples, including *Acer negundo*.

The Box Elder floodplain forest found closest to the West Branch of the Little Conestoga Creek is an early successional palustrine community that arises from either natural or anthropogenic (as in this case) floodplain disturbances [95]. It is dominated by *Acer negundo* (Box Elder) and *Platanus occidentalis* (American Sycamore), as well as other *Acer* spp. (maples) and *Salix* spp. (willows) [94], [95]. These three forest classifications (Red Oak-Sugar Maple Forest, northeastern modified successional forest, and Box Elder forest) contain many of the same taxa, and taken together, they represent all the major canopy-forming trees identified in the field around DM as well as the majority of the subcanopy species present.

### Paleocommunity Interpretation

The pre-settlement flora from Denlingers Mill represent an ecologically diverse mix of species that was previously not known to have existed in association in the pre-colonial forests of the region, ranging from herbaceous wetland plants to hydrophylic and upper slope woody taxa. Qualitative abundance data indicate that *Fagus grandifolia* (American Beech) and *Quercus* spp. (Red and White Oaks), all facultative upland, were the predominant species in the ancient old-growth forest. Because contemporary forest classifications are based on dominant species, the pre-settlement upland forest can be considered analogous to a modern Red Oak-American Beech mixed hardwood forest, which is a broadly defined community that occurs on mesic sites and is variable in composition [94]. The dominant canopy species of this modern-day forest classification are *Quercus* spp., *Fagus grandifolia*, and *Acer rubrum*, while typical subcanopy species are *Betula lenta*, *Liriodendron tulipifera*, and *Ostrya virginiana*. The pre-settlement forest at DM was most likely similar to contemporary Red Oak-American Beech forests; these contain all the facultative upland species found in the DM subfossil deposit except *Acer spicatum,* which is typically a subcanopy species, and *Castanea dentata*, which is no longer included in forest classifications.

Although the upland forest is the best recorded here, this fossil assemblage probably represents other communities as well, similar to those previously described in preliminary reports (Table 5). Big Spring Run, also located in Lancaster County, and White Clay Creek, in neighboring Chester County, are two sites highly influenced by milldams. Both locations now support altered, incised, single-channel streams lined by weedy vegetation, in contrast to the many small anabranching channels bordered by hydrophylic trees, shrubs, and sedges hypothesized to have prevailed in the past.

Macrofossil fruits and seeds from Big Spring Run [8], and fragmentary leaf and fruit macrofossils from White Clay Creek [11], [12], have supplied information about the pre-settlement herbaceous wetland and lowland tree components, respectively. As mentioned above, the valley-bottom tussock sedge wetlands studied at Big Spring Run were composed mainly of *Carex* spp., *Scirpus* spp., and *Eleocharis* spp. The subfossil leaf assemblage from White Clay Creek contained mostly facultative and facultative wetland species such as *Acer rubrum*, *Acer negundo, Alnus serrulata*, and *Salix* spp., as well as some facultative upland species including *Fagus grandifolia, Quercus* spp., and *Liriodendron tulipifera*. Although no *Fraxinus* material was found at White Clay Creek, these fossils were provisionally interpreted as representing a Red Maple-Black Ash deciduous swamp forest [12]. This forest type commonly occurs on floodplains, usually with a sedge-dominated (*Carex* spp.) understory, and is sensitive to changes in hydrology [95]. In addition to the two dominant species *Acer rubrum* and *Fraxinus nigra*, this community frequently contains *Betula* spp., *Quercus* spp. and *Salix* spp. [94], all of which are found in the subfossil assemblage at DM (Table 5). The results of these two previous studies contain overlapping tree taxa such as *A. rubrum* and *L. tulipifera*, suggesting that the two hypothesized pre-settlement communities were not isolated occurrences but were instead regionally widespread.

**Figure 17:**
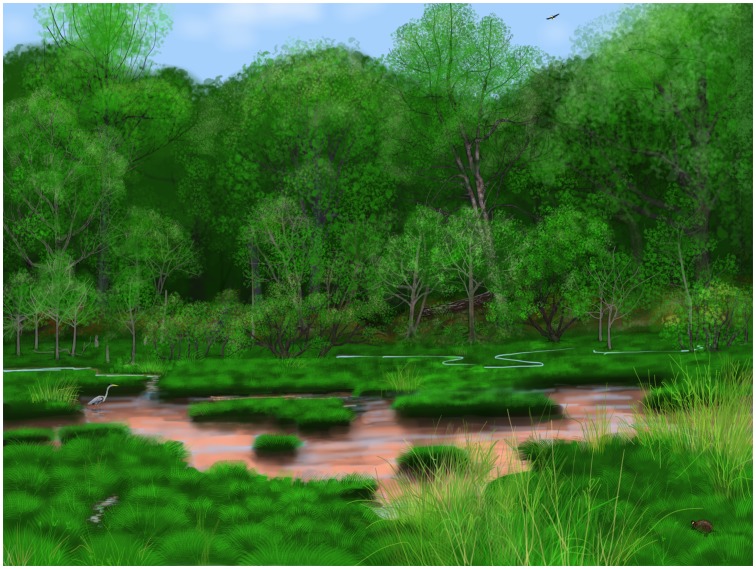
Artist's reconstruction of the pre-settlement riparian, transitional, and upslope landscape as here interpreted using plant macrofossils from southeastern Pennsylvania (see text). The valley-bottom foreground consists of palustrine, persistent, emergent wetlands with tussock sedge vegetation, including *Carex* spp., *Eleocharis* spp., and *Scirpus* spp. [8]. Lower slopes support a Red Maple-Black Ash deciduous swamp forest, described here and in [12]. Other components of this community include Hazel Alder (*Alnus serrulata*) and various species of willow (*Salix* spp.). The upper slope forest community in the background is a Red Oak-American Beech mixed hardwood forest dominated by various species of red and white oaks (*Quercus* spp.), American Beech (*Fagus grandifolia)*, and Sweet Birch *(Betula lenta)*. Other community constituents include Eastern Hophornbeam (*Ostrya virginiana*), American Chestnut (*Castanea dentata*), Mountain Maple (*Acer spicatum*), American Sycamore (*Platanus occidentalis*), and Tulip Tree (*Liriodendron tulipifera*). Artwork: Rebecca Wilf.

Integrating the results from this study, White Clay Creek, and Big Spring Run, we propose here that the pre-settlement landscape in the area of Lancaster and Chester counties consisted of at least three distinct communities with a continuum of overlapping species (Fig. 17). These communities graded from saturated tussock-sedge wetlands represented by remnant herbaceous material in valley-bottoms, to a transitional maple and ash floodplain swamp forest community fringing the wetlands evidenced at DM by *Acer rubrum, Fraxinus nigra, Platanus occidentalis*, and *Salix* spp., and then into an Oak and Beech-dominated mixed hardwood forest on valley slopes shown at DM by relatively abundant *Fagus grandifolia*, *Quercus* spp., and *Betula lenta* leaf macrofossils. Less abundant upland community constituents included *Acer spicatum*, *Castanea dentata*, and *Ostrya virginiana*. Although the contemporary riparian and upslope forest at Denlingers Mill today has some species in common with the subfossil assemblage, including *Quercus* spp., *F. grandifolia*, *B. lenta*, and *P. occidentalis*, the modern forest at this site is overwhelmingly dominated by *Acer negundo* and *Acer saccharum*, which are characteristically disturbance and successional species, thus highlighting the turnover in forest composition and structure due to anthropogenic activities.

## Conclusions

Subfossil leaves from Denlingers Mill refine and enhance our understanding of a past complex and dynamic landscape by providing insight into previously unknown, pre-European settlement valley margin and upslope hardwood tree communities. Comparison of the riparian fossilized old-growth and modern secondary forests from Denlingers Mill illustrates the lasting detrimental effects of colonial-era modifications to the Piedmont landscape, especially because the secondary forests that dominate the landscape today have very different structure and composition and are ecologically functionally inferior to the ancient forests. Additionally, this work demonstrates that leaf deposits contain information not available from other plant macrofossil sources, especially in revealing local, temporally constrained signals. Leaf fossils can and should be more often used, together with all other available fossil materials, in order to facilitate more accurate paleoecological reconstructions in relatively recent strata, as well as in deep-time work as already widely practiced.

Ultimately, the floral communities described here can be used not only to help understand the forests in which both Native American and European settlement occurred, but also to inform stream restoration decisions regarding the re-establishment of stream-accessible floodplains and self-sustaining wetland, riparian, and upland communities along Piedmont reaches. Moreover, hardwood trees in the riparian buffer zone are important to both aquatic and terrestrial wildlife because leaf litter provides sustenance for in-stream biota, such as macroinvertebrates, and, in conjunction with well-vegetated wetlands, valley margin and slope hardwood forests provide prime habitat for terrestrial organisms [34]. Increased restoration success based on expanded knowledge about both past and present plant communities is not only environmentally advantageous, it is also economically beneficial considering that restoration of riparian buffers and associated wetlands commonly costs hundreds of thousands of dollars per stream mile at present, often with unsuccessful results [97]. Additionally, regional restoration efforts may have potential regarding future climate-change mitigation because wetlands act as major carbon sinks.

## Supporting Information

Table S1 Sample inventory.
(DOCX)

## References

[1] Gottschalk LC (1945) Effects of soil erosion on navigation in upper Chesapeake Bay. Geogr Rev 35: 219–238.

[2] Foster DR, Motzkin G, Slater B (1998) Land-use history as long-term broad-scale disturbance: regional forest dynamics in central New England. Ecosystems 1: 96–119.

[3] Cogbill CV (2000) Vegetation of the presettlement forests of northern New England and New York. Rhodora 102: 250–276.

[4] Walter RC, Merritts DJ (2008) Natural streams and the legacy of water-powered mills. Science 319: 299–304. 18202284 10.1126/science.1151716

[5] Keeton WS, Kraft CE, Warren DR (2007) Mature and old-growth riparian forests: structure, dynamics, and effects on Adirondack stream habitats. Ecol Appl 17: 852–868. 17494402 10.1890/06-1172

[6] Kemp WM, Boynton WR, Adolf JE, Boesch DF, Boicourt WC, et al (2005) Eutrophication of Chesapeake Bay: historical trends and ecological interactions. Mar Ecol Prog Series 303: 1–29.

[7] Richardson DM, Holmes PM, Esler KJ, Galatowitsch SM, Stromberg JC, et al (2007) Riparian vegetation: degradation, alien plant invasions, and restoration prospects. Divers Distrib 13: 126–139.

[8] Voli M, Merritts D, Walter R, Ohlson E, Datin K, et al (2009) Preliminary reconstruction of a pre-European settlement valley bottom wetland, southeastern Pennsylvania. Water Res Impact 11: 11–13.

[9] Hilgartner WB, Brush GS (2006) Prehistoric habitat stability and post-settlement habitat change in a Chesapeake Bay freshwater tidal wetland, USA. Holocene 16: 479–494.

[10] Hilgartner W, Merritts D, Walter R, Rahnis M (2010) Pre-settlement habitat stability and post-settlement burial of a tussock sedge (*Carex stricta*) wetland in a Maryland Piedmont river valley. Ecol Soc Am Ann Meet, Pittsburgh, Pennsylvania, abstract: 8–69.

[11] Miller CL (2010) A pre-settlement flora of White Clay Creek, PA. Geol Soc Am Ann Meet, Denver, Colorado, Abstr Prog 42: 606.

[12] Miller CL (2011) Lessons from soggy leaves: a pre-settlement flora from White Clay Creek, Chester County, Pennsylvania. Masters thesis. The Pennsyvania State University.

[13] Spicer RA (1989) The formation and interpretation of plant fossil assemblages. Adv Bot Res 16: 95–191.

[14] Burnham RJ (1993) Reconstructing richness in the plant fossil record. Palaios 8: 376–384.

[15] Work PT, Semken HA, Baker RG (2005) Pollen, plant macrofossils and microvertebrates from mid-Holocene alluvium in east-central Iowa, USA: comparative taphonomy and paleoecology. Palaeogeogr Palaeoclimatol Palaeoecol 223: 204–221.

[16] Chumbley CA (1989) Late-glacial and Holocene vegetation of the Roberts Creek Basin, northeast Iowa. PhD Dissert. University of Iowa.

[17] Baker RG, Chumbley CA, Witinok PM, Kim HK (1990) Holocene vegetational changes in eastern Iowa. J Iowa Acad Sci 97: 167–177.

[18] Miller BB, Graham RW, Morgan AV, Miller NG, McCoy WD, et al (1994) A biota associated with Matuyama-age sediments in west-central Illinois. Quatern Res 41: 350–365.

[19] Birks HH, Birks HJB (2000) Future uses of pollen analysis must include plant macrofossils. J Biogeogr 27: 31–35.

[20] Birks HH, Mathewes RW (1978) Studies in the vegetational history of Scotland V. Late Devensian and early Flandrian pollen and macrofossil stratigraphy at Abernethy Forest, Inverness-Shire. New Phytol 80: 455–484.

[21] Wing SL, DiMichele WA (1995) Conflict between local and global changes in plant diversity through geological time. Palaios 10: 551–564.

[22] Davies-Vollum KS, Wing SL (1998) Sedimentological, taphonomic, and climatic aspects of Eocene swamp deposits (Willwood Formation, Bighorn Basin, Wyoming). Palaios 13: 28–40.

[23] Behrensmeyer AK, Kidwell SM, Gastaldo RA (2000) Taphonomy and paleobiology. Paleobiology 26(4S): 103–147.

[24] Costa JE (1975) Effects of agriculture on erosion and sedimentation in the Piedmont province, Maryland. Geol Soc Am Bull 86: 1281–1286.

[25] Lemon JT (2002) The best poor man’s country: early southeastern Pennsylvania. Baltimore: Johns Hopkins University Press. 295 p.

[26] Middleton AP (1953) Tobacco Coast: a maritime history of Chesapeake Bay in the colonial era. Richmond: Whittet and Shepperson. 482 p.

[27] Hartfranft JL, Merritts DJ, Walter RC, Rahnis M (2011) The Big Spring Run restoration experiment: policy, geomorphology, and aquatic ecosystems in the Big Spring Run watershed, Lancaster County, PA. Int J Environ Cult Econ Soc Sustain 24: 24–30.

[28] Simon A (1989) The discharge of sediment in channelized alluvial streams. Water Res Bull 25: 1177–1188.

[29] Merritts D, Walter R, Rahnis M, Hartranft J, Cox S, et al (2011) Anthropocene streams and base-level controls from historic dams in the unglaciated mid-Atlantic region, USA. Phil Trans R Soc A 369: 976–1009. 21282157 10.1098/rsta.2010.0335

[30] Shafroth PB, Stromberg JC, Patten DT (2002) Riparian vegetation response to altered disturbance and stress regimes. Ecol Appl 12: 107–123.

[31] Stanley EH, Doyle DW (2003) Trading off: the ecological effects of dam removal. Front Ecol Environ 1: 15–22.

[32] Gutshall M (2004) Back to the future: stream corridor restoration and some new uses for old floodplains. Landstudies, Inc. Report. Available: http://www.landstudies.com?BacktotheFuture.pdf. Accessed 2 February 2012.

[33] Doyle MW, Stanley EH, Orr CH, Selle AR, Sethi SA, et al (2005) Stream ecosystem response to small dam removal: lessons from the heartland. Geomorphology 71: 227–244.

[34] Gutshall MA, Oberholtzer WL (2011) Floodplain restoration: basics, benefits, and practical applications. Int J Environ Cult Econ Soc Sustain 24: 14–23.

[35] Lorimer CG, Frelich LE (1994) Natural disturbance regimes in old-growth northern hardwoods. J Forest 92: 33–38.

[36] Bilby RE, Likens GE (1980) Importance of organic debris dams in the structure and function of stream ecosystems. Ecology 61: 1107–1113.

[37] Douglas I (1991) Sediment transfer and siltation. In: Turner BL, Clark WC, Kates RW, Richards JF, Mathews JT, Meyer WB, editors. The earth as transformed by human action: global and regional changes in the biosphere over the past 300 years. Cambridge: Cambridge University Press. 215–234.

[38] Denevan WM (1992) The pristine myth: the landscape of the Americas in 1492. Ann Assoc Am Geogr 82: 369–385.

[39] Springer GS, White DM, Rowe HD, Mihimdukulasooriya LN, Cheng H, et al (2010) Multiproxy evidence from caves of Native Americans altering the overlying landscape during the late Holocene of east-central North America. Holocene 20: 275–283.

[40] Baker RG, Schwert DP, Bettis EA III, Chumbley CA (1993) Impact of Euro-American settlement on a riparian landscape in northeast Iowa, midwestem USA: an integrated approach based on historical evidence, floodplain sediments, fossil pollen, plant macrofossils and insects. Holocene 3: 314–323.

[41] Black BA, Ruffner CM, Abrams MD (2006) Native American influences on the forest composition of the Allegheny Plateau, northwest Pennsylvania. Can J Forest Res 36: 1266–1275.

[42] Stinchcomb GE, Messner TC, Driese SG, Nordt LC, Stewart RM (2011) Pre-colonial (A.D. 1100–1600) sedimentation related to prehistoric maize agriculture and climate change in eastern North America. Geology 39: 363–366.

[43] Asch Sidell N (2008) The impact of maize-based agriculture on prehistoric plant communities in the northeast. In: New York: State Mus Bull Hart, J.P, editor. Current northeast paleoethnobotany II. 512: 29–52.

[44] Palmer MA, Bernhardt ES, Allan JD, Lake PS, Alexander G, et al (2005) Standards for ecologically successful river restoration. J Appl Ecol 42: 208–217.

[45] Bennion H, Battarbee RW, Sayer CD, Simpson GL, Davidson TA (2011) Defining reference conditions and restoration targets for lake ecosystems using palaeolimnology: a synthesis. J Paleolimnol 45: 533–544.

[46] Walter R, Merritts D, Rahnis M (2007) Estimating volume, nutrient content, and rates of stream bank erosion of legacy sediment in the Piedmont and Valley and Ridge physiographic provinces, southeastern and central PA. Report to the Pennsylvania Department of Environmental Protection, Lancaster, Pennsylvania, 40 p.

[47] Merritts D, Walter R, Rahnis M (2010) Water-powered milling and its legacy as a source of suspended sediment to the Susquehanna River and Chesapeake Bay. Susquehanna River Basin Commission, Harrisburg, PA. Available: http://www.srbc.net/stateofsusq2010/documents/legacysedimentsfeaturearticle.pdf. Accessed 23 October 2012.

[48] Merritts D, Walter R, Rahnis MA (2010) Sediment and nutrient loads from stream corridor erosion along breached millponds. Available: https://edisk.fandm.edu/michael.rahnis/outgoing/DEP/DEP_REPORT_TEXT.pdf. Accessed 23 October 2012.

[49] Gellis AC, Hupp CR, Pavich MJ, Landwehr JM, Banks WSL, et al. (2009) Sources, transport, and storage of sediment at selected sites in the Chesapeake Bay watershed. Scientific Investigations Report 2008–5186, 95 p. Available: http://pubs.usgs.gov/sir/2008/5186/. Accessed 22 October 2012.

[50] Schenk ER, Hupp CR (2009) Legacy effects of colonial millponds on floodplain sedimentation, bank erosion, and channel mophology, mid-Atlantic, USA. J Am Water Res Assoc 45: 597–606.

[51] Moss JH, Kochel CR (1978) Unexpected geomorphic effects of the Hurricane Agnes storm and flood, Conestoga drainage basin, southeastern Pennsylvania. J Geol 86: 1–11.

[52] Merritts D, Walter R (2003) Colonial mill ponds of Lancaster County, Pennsylvania as a major source of sediment pollution to the Susquehanna River and Chesapeake Bay. SEFOP Guidebook. Available: http://facewebsites.com/spoommidatlantic/site_files/editor_files/image/file/Milling%20History/ColonialMillSediment.pdf. Accessed: 23 October 2012.

[53] Merritts DJ, Walter RC, Rahnis M (2013) The rise and fall of Mid-Atlantic streams: millpond sedimentation, milldam breaching, channel incision, and stream bank erosion. Geol Soc Am Rev Engin Geol XXI: 183–203.

[54] Pizzuto J, O'Neal M (2009) Increased mid-twentieth century riverbank erosion rates related to the demise of mill dams, South River, Virginia. Geology 37: 19–22.

[55] Orr CH (2002) Patterns of removal and ecological response: a study of small dams in Wisconsin. Masters Thesis. University of Wisconsin.

[56] Palmer M, Allan JD, Meyer J, Bernhardt ES (2007) River restoration in the twenty-first century: data and experiential knowledge to inform future efforts. Restor Ecol 15: 472–481.

[57] Niemitz J, Haynes C, Lasher G (2012) Legacy sediments and historic land use: chemostratigraphic evidence for excess nutrient and heavy metal sources and remobilization. Geology 41: 47–50.

[58] Stanley EH, Doyle MW (2002) A geomorphic perspective on nutrient retention following dam removal. Bioscience 52: 693–701.

[59] Boynton WR, Garber JH, Summers R, Kemp WM (1995) Inputs, transformations, and transport of nitrogen and phosphorus in Chesapeake Bay and selected tributaries. Estuaries 18: 285–314.

[60] Waters TF (1995) Sediment in streams: sources, biological effects, and control. Bethesda: American Fisheries Society Monograph. 251 p.

[61] Pennock JR, Sharp JH, Schroeder WW (1994) What controls the expression of estuarine eutrophication? Case studies of nutrient enrichment in the Delaware Bay and Mobile Bay estuaries, USA. In: Dyer KR, Orth RJ, editors. Changes in fluxes in estuaries: implications from science to management. Fredensborg, Denmark: Olsen & Olsen. 139–146.

[62] Boesch DF, Brinsfield RB, Magnien RE (2001) Chesapeake Bay eutrophication: scientific understanding, ecosystem restoration, and challenges for agriculture. J Environ Qual 30: 303–320. 11285890 10.2134/jeq2001.302303x

[63] Orth RJ, Moore KA (1983) Chesapeake Bay: an unprecedented decline in submerged aquatic vegetation. Science 222: 51–53. 17810089 10.1126/science.222.4619.51

[64] FISRWG (Federal Interagency Stream Research Working Group) (2008) Stream corridor restoration: principles, processes, and practices. Natural Resources Conservation Services, Washington D.C. Available: http://www.nrcs.usda.gov/wps/portal/nrcs/detailfull/national/water/manage/?&cid=stelprdb1043244. Accessed 23 October 2012.

[65] Keller EA, Swanson FJ (1979) Effects of large organic material on channel form and fluvial processes. Earth Surf Proc 4: 361–380.

[66] Ferguson DK (1985) The origin of leaf-assemblages – new light on an old problem. Rev Palaeobot Palynol 46: 117–188.

[67] Burnham RJ, Wing SL, Parker GG (1992) The reflection of deciduous forest communities in leaf litter: implications for autochthonous litter assemblages from the fossil record. Paleobiology 18: 30–49.

[68] Fenneman NM (1938) Physiography of eastern United States. New York: McGraw Hill.

[69] Danzeglocke U, Jöris O, Weninger B (2011) CalPal –2007online. Available: http://www.calpal-online.de/. Accessed 15 July 2012.

[70] Ellis D, Daly DC, Hickey LJ, Johnson KR, Mitchell JD, et al.. (2009) Manual of leaf architecture – morphological description and categorization of dicotyledonous and net-veined monocotyledonous angiosperms. Ithaca: Cornell University Press. 190 p.

[71] Wilf P (1997) When are leaves good thermometers? A new case for leaf margin analysis. Paleobiology 23: 373–390.

[72] Hardin JW (1979) Atlas of foliar surface features in woody plants, I. Vestiture and trichome types of eastern North American *Quercus* . Bull Torrey Bot Club 106: 313–325.

[73] Hardin JW, Johnson GP (1985) Atlas of foliar surface features in woody plants, VIII. *Fagus* and *Castanea* (Fagaceae) of eastern North America. Bull Torrey Bot Club 112: 11–20.

[74] Hardin JW, Bell JM (1986) Atlas of foliar surface features in woody plants, IX. Betulaceae of eastern United States. Brittonia 38: 133–144.

[75] Barclay RS, Wilf P, Dilcher DL, McElwain JC (2012) The Cuticle Database Project, version 1.1. University Park: The Earth and Environmental Systems Institute of Pennsylvania State University. Available: http://cuticledb.eesi.psu.edu/. Accessed 20 October 2012.

[76] Mann ME (2002) Little Ice Age. In: Munn T, editor. Encyclopedia of Global Environmental Change. Chichester: John Wiley & Sons, Ltd. 504–509.

[77] R Development Core Team (2010) R: A language and environment for statistical computing. Vienna: R Foundation for Statistical Computing. Available: http://www.R-project.org. Accessed 20 June 2012.

[78] United States Department of Agriculture, Natural Resources Conservation Service (2011) The PLANTS Database. Baton Rouge: National Plant Data Center. Available: http://www.plants.usda.gov. Accessed 30 May 2012.

[79] Fernald ML (1950) Gray’s manual of botany, 8th (Centennial) Edition. New York: The American Book Company. 1632 p.

[80] Little EL (1980) The Audubon Society field guide to North American trees, eastern region. New York: Alfred A. Knopf Publishing. 716 p.

[81] Rhoads AF, Block TA (2007) The plants of Pennsylvania, an illustrated manual 2nd edition. Philadelphia: University of Pennsylvania Press.

[82] Virginia Tech Forest Resources and Environmental Conservation, Tree ID Database (2010) Blacksburg: Virginia Tech Department of Forest Resources and Environmental Conservation. Available: http://www.dendro.cnre.vt.edu/dendrology/factsheets.cfm. Accessed 15 June 2012.

[83] Rhoads AF, Block TA (2005) Trees of Pennsylvania: a complete reference guide. Philadelphia: University of Pennsylvania Press.

[84] Sibley DA (2009) The Sibley guide to trees. New York: Alfred A. Knopf Publishing. 464 p.

[85] Gleason HA, Cronquist A (1963) Manual of vascular plants of northeastern United States and adjacent Canada. Boston: Willard Grant Press. 810 p.

[86] eFloras (2008): Missouri Botanical Garden, St. Louis, Missouri and Harvard University Herbaria, Cambridge, MA. Online database. Available: http://www.efloras.org/. Accessed 23 October 2012.

[87] Coladonato M (1991) *Fagus grandifolia* In: Fire Effects Information System, [Online]. U.S. Department of Agriculture, Forest Service, Rocky Mountain Research Station, Fire Sciences Laboratory (Producer). Available: http://www.fs.fed.us/database/feis/. Accessed 23 October 2012.

[88] McQuilkin RA (1992) *Quercus palustris* L. In: Burns RM, Honkala B, editors. Silvics of North America Volume 2: Hardwoods. Washington D.C.: U.S. Department of Agriculture Forest Service. 1366–1377. Available: http://www.na.fs.fed.us/pubs/silvics_manual/volume_2/silvics_v2.pdf. Accessed 3 April 2013.

[89] Sander IL (1990) *Quercus rubra* L. In: Burns RM, Honkala B, editors. Silvics of North America Volume 2: Hardwoods. Washington, DC: U.S. Department of Agriculture, Forest Service. 1401–1414.

[90] Johnson PS (1992) *Quercus coccinea* L. In: Burns RM, Honkala B, editors. Silvics of North America Volume 2: Hardwoods. Washington D.C.: U.S. Department of Agriculture, Forest Service. 1219–1226. Available: http://www.na.fs.fed.us/pubs/silvics_manual/volume_2/silvics_v2.pdf. Accessed 3 April 2013.

[91] Hickey LJ, Wolfe JA (1975) The bases of angiosperm phylogeny: vegetative morphology. Ann Mo Bot Gard 62: 538–589.

[92] Argus GW (2006) Guide to the identification of *Salix* (willow) in Illinois, Indiana, Ohio, and Pennsylvania. Ottawa: Canadian Museum of Nature. Available: http://aknhp.uaa.alaska.edu/wp-content/uploads/2011/02/GuideSalixMIDWEST-24APR2006.pdf. Accessed: 15 September 2012.

[93] Carpenter RJ, Hill RS, Jordan GJ (2005) Leaf cuticular morphology links Platanaceae and Proteaceae. Int J Plant Sci 166: 843–855.

[94] Fike J (1999) Terrestrial and palustrine plant communities of Pennsylvania. Pennsylvania Dept. of Conservation and Natural Resources, Harrisburg, The Nature Conservancy, Middletown, and Western Pennsylvania Conservancy, Pittsburgh. 86 p. Available: http://www.naturalheritage.state.pa.us/fikebook/terrestrial_plant_book.pdf. Accessed 23 October 2012.

[95] Westervelt K, Largay E, Coxe R, McAvoy W, Perles S, et al. (2006) A guide to the natural communities of the Delaware estuary: Version 1. Arlington: Nature Serve. Available: http://www.delawareestuary.org/NVCS/Prnt_Guide%20to%20the%20Natural%20Communities%20of%20DE%20Estuary_v1.pdf. Accessed 23 October 2012.

[96] Fleming GP, Van Alstine NE (1999) Plant communities and floristic features of sinkhole ponds and seepage wetlands in southeastern Augusta County, Virginia. Banisteria 13: 67–94.

[97] Kenney MA, Wilcock PR, Hobbs BF, Flores NE, Martınez DC (2012) Is urban stream restoration worth it? J Am Water Res Assoc 48: 603–615.

