# Supplementary material for: Subfossil Leaves Reveal a New Upland Hardwood Component of the Pre-European Piedmont Landscape, Lancaster County, Pennsylvania
Source: PLoS One. 2013 Nov 13;8(11):e79317. doi: 10.1371/journal.pone.0079317 (PMC3827356; doi:10.1371/journal.pone.0079317)
Supplement: Table S1 — Sample inventory. (DOCX) [file pone.0079317.s001.docx]

| **Table S1. Sample inventory.** |  |  |
| --- | --- | --- |
| **Identification** | **Sample #** | **EMS #** |
| *Salix* sp. | DM33 | EMS419501 |
| *Acer spicatum* | DM9 | EMS419502 |
| *Acer rubrum* | DM32 | EMS419503 |
| *Acer rubrum* | DM5 | EMS419504 |
| *Acer rubrum* | DM13 | EMS419505 |
| *Platanus occidentalis* | DM42 | EMS419506 |
| *Fraxinus nigra* | DM8 (samara) | EMS419507 |
| *Quercus* cf. *alba* | DM26 | EMS419508 |
| *Quercus* cf. *alba* | DM27 | EMS419509 |
| *Quercus* cf. *alba* | DM15 | EMS419510 |
| *Quercus* cf. *alba* | DM29 | EMS419511 |
| *Quercus* cf. *alba* | DM36 | EMS419512 |
| *Quercus* cf. *alba* | DM37 | EMS419513 |
| *Quercus* cf. *alba* | DM38 | EMS419514 |
| *Quercus* cf. *alba* | DM39 | EMS419515 |
| *Quercus* subgenus *Lobatae* | DM14 | EMS419516 |
| *Quercus* subgenus *Lobatae* | DM91 | EMS419517 |
| *Quercus* subgenus *Lobatae* | DM99 | EMS419518 |
| *Quercus* subgenus *Lobatae* | DM22 | EMS419519 |
| *Quercus* subgenus *Lobatae* | DM28 | EMS419520 |
| *Quercus* subgenus *Lobatae* | DM92 | EMS419521 |
| *Quercus* subgenus *Lobatae* | DM77 | EMS419522 |
| *Quercus* subgenus *Lobatae* | DM78 | EMS419523 |
| *Quercus* subgenus *Lobatae* | DM20 | EMS419524 |
| *Quercus* subgenus *Lobatae* | DM90 | EMS419525 |
| *Quercus* subgenus *Lobatae* | DM80 | EMS419526 |
| *Quercus* subgenus *Lobatae* | DM89 | EMS419527 |
| *Quercus* subgenus *Lobatae* | DM102 | EMS419528 |
| *Quercus* subgenus *Lobatae* | DM94 | EMS419529 |
| *Quercus* subgenus *Lobatae* | DM105 | EMS419530 |
| *Quercus* subgenus *Lobatae* | DM107 | EMS419531 |
| *Quercus* subgenus *Lobatae* | DM17 | EMS419532 |
| *Quercus* subgenus *Lobatae* | DM23 | EMS419533 |
| *Quercus* subgenus *Lobatae* | DM30 | EMS419534 |
| *Quercus* subgenus *Lobatae* | DM79 | EMS419535 |
| *Quercus* subgenus *Lobatae* | DM81 | EMS419536 |
| *Quercus* subgenus *Lobatae* | DM82 | EMS419537 |
| *Quercus* subgenus *Lobatae* | DM83 | EMS419538 |
| *Quercus* subgenus *Lobatae* | DM84 | EMS419539 |
| *Quercus* subgenus *Lobatae* | DM85 | EMS419540 |

| *Quercus* subgenus *Lobatae* | DM86 | EMS419541 |
| --- | --- | --- |
| *Quercus* subgenus *Lobatae* | DM87 | EMS419542 |
| *Quercus* subgenus *Lobatae* | DM88 | EMS419543 |
| *Quercus* subgenus *Lobatae* | DM93 | EMS419544 |
| *Quercus* subgenus *Lobatae* | DM95 | EMS419545 |
| *Quercus* subgenus *Lobatae* | DM96 | EMS419546 |
| *Quercus* subgenus *Lobatae* | DM97 | EMS419547 |
| *Quercus* subgenus *Lobatae* | DM98 | EMS419548 |
| *Quercus* subgenus *Lobatae* | DM100 | EMS419549 |
| *Quercus* subgenus *Lobatae* | DM101 | EMS419550 |
| *Quercus* subgenus *Lobatae* | DM103 | EMS419551 |
| *Quercus* subgenus *Lobatae* | DM104 | EMS419552 |
| *Quercus* subgenus *Lobatae* | DM106 | EMS419553 |
| *Quercus* subgenus *Lobatae* | DM108 (acorn) | EMS419554 |
| *Castanea dentata* | DM6 | EMS419555 |
| *Fagus grandifolia* | DM1 | EMS419556 |
| *Fagus grandifolia* | DM50 | EMS419557 |
| *Fagus grandifolia* | DM48 | EMS419558 |
| *Fagus grandifolia* | DM21 | EMS419559 |
| *Fagus grandifolia* | DM25 | EMS419560 |
| *Fagus grandifolia* | DM31 | EMS419561 |
| *Fagus grandifolia* | DM44 | EMS419562 |
| *Fagus grandifolia* | DM45 | EMS419563 |
| *Fagus grandifolia* | DM46 | EMS419564 |
| *Fagus grandifolia* | DM47 | EMS419565 |
| *Fagus grandifolia* | DM49 | EMS419566 |
| *Fagus grandifolia* | DM51 | EMS419567 |
| *Fagus grandifolia* | DM52 | EMS419568 |
| *Fagus grandifolia* | DM53 | EMS419569 |
| *Fagus grandifolia* | DM54 | EMS419570 |
| *Fagus grandifolia* | DM55 | EMS419571 |
| *Fagus grandifolia* | DM56 | EMS419572 |
| *Fagus grandifolia* | DM57 | EMS419573 |
| *Fagus grandifolia* | DM58 | EMS419574 |
| *Fagus grandifolia* | DM59 | EMS419575 |
| *Fagus grandifolia* | DM60 | EMS419576 |
| *Fagus grandifolia* | DM61 | EMS419577 |
| *Betula lenta* | DM2 | EMS419578 |
| *Betula lenta* | DM3 | EMS419579 |
| *Betula lenta* | DM4 | EMS419580 |
| *Betula lenta* | DM11 | EMS419581 |
| *Betula lenta* | DM12 | EMS419582 |
| *Betula lenta* | DM16 | EMS419583 |

| *Betula lenta* | DM18 | EMS419584 |
| --- | --- | --- |
| *Betula lenta* | DM19 | EMS419585 |
| *Betula lenta* | DM64 | EMS419586 |
| *Betula lenta* | DM65 | EMS419587 |
| *Betula lenta* | DM66 | EMS419588 |
| *Betula lenta* | DM67 | EMS419589 |
| *Betula lenta* | DM68 | EMS419590 |
| *Betula lenta* | DM69 | EMS419591 |
| *Betula lenta* | DM70 | EMS419592 |
| *Ostrya virginiana* | DM40 | EMS419593 |
| *Ostrya virginiana* | DM63 | EMS419594 |
| *Ostrya virginiana* | DM7 (seed w/bract) | EMS419595 |
| Unidentified sample | DM10 | EMS419596 |
| Unidentified sample | DM24 | EMS419597 |
| Unidentified sample | DM35 | EMS419598 |
| Unidentified sample | DM34 | EMS419599 |
| Unidentified sample | DM62 | EMS419600 |
| Unidentified sample | DM71 | EMS419601 |
| Unidentified sample | DM72 | EMS419602 |
| Unidentified sample | DM73 | EMS419603 |
| Unidentified sample | DM74 | EMS419604 |
| Unidentified sample | DM75 | EMS419605 |
| Unidentified sample | DM76 | EMS419606 |
| Unidentified sample | DM41 | EMS419607 |
| Unidentified sample | DM43 | EMS419608 |
| Unidentified sample | DM109 | EMS419609 |
| Unidentified sample | DM110 | EMS419610 |
| Unidentified sample | DM111 | EMS419611 |
| Unidentified sample | DM112 | EMS419612 |
| Unidentified sample | DM113 | EMS419613 |
| Unidentified sample | DM114 | EMS419614 |
| Unidentified sample | DM115 | EMS419615 |
| Unidentified sample | DM116 | EMS419616 |
| Unidentified sample | DM117 | EMS419617 |
| Unidentified sample | DM118 | EMS419618 |
| Unidentified sample | DM119 | EMS419619 |
| Unidentified sample | DM120 | EMS419620 |
| Unidentified sample | DM121 | EMS419621 |
| Unidentified sample | DM122 | EMS419622 |
| Unidentified sample | DM123 | EMS419623 |
| Unidentified sample | DM124 | EMS419624 |
| Unidentified sample | DMP1 | EMS419625 |
| Unidentified sample | DMP2 | EMS419626 |
